# Enhanced aquila optimizer for global optimization and data clustering

**DOI:** 10.1038/s41598-025-95888-w

**Published:** 2025-04-16

**Authors:** Laith Abualigah, Saleh Ali Alomari, Mohammad H. Almomani, Raed Abu Zitar, Hazem Migdady, Kashif Saleem, Aseel Smerat, Vaclav Snasel, Amir H. Gandomi

**Affiliations:** 1 Computer Science Department, Al al-Bayt University, Mafraq, 25113 Jordan; 2 Faculty of Information Technology, Jadara University, Irbid, 21110 Jordan; 3 Department of Mathematics, Facility of Science, The Hashemite University, P.O Box 330127, Zarqa, 13133 Jordan; 4 Faculty of Engineering and Computing, Liwa College, Abu Dhabi, United Arab Emirates; 5 CSMIS Department, Oman College of Management and Technology, 320 Barka, Oman; 6 Department of Computer Science & Engineering, College of Applied Studies & Community Service, King Saud University, 11362 Riyadh, Saudi Arabia; 7 Faculty of Educational Sciences, Al-Ahliyya Amman University, Amman, 19328 Jordan; 8 Centre for Research Impact & Outcome, Chitkara University Institute of Engineering and Technology, Chitkara University, Rajpura, Punjab 140401 India; 9 Computer Technologies Engineering, Mazaya University College, Nasiriyah, Iraq; 10 Faculty of Electrical Engineering and Computer Science, VŠB-Technical University of Ostrava, 70800 Poruba-Ostrava, Czech Republic; 11 Faculty of Engineering and Information Technology, University of Technology Sydney, Ultimo, NSW 2007 Australia; 12 School of Engineering and Technology, Sunway University Malaysia, Petaling Jaya 27500, Malaysia; 13 University Research and Innovation Center (EKIK), Óbuda University, 1034 Budapest, Hungary; 14 Department of Computer Science, Khazar University, Baku, Azerbaijan

**Keywords:** Aquila optimizer, Meta-heuristics optimization algorithms, Opposition-based learning, Data clustering problems, Optimization problems, Energy science and technology, Engineering, Mathematics and computing

## Abstract

The Aquila Optimizer (AO) is a newly proposed, highly capable metaheuristic algorithm based on the hunting and search behavior of the Aquila bird. However, the AO faces some challenges when dealing with high-dimensional optimization problems due to its narrow exploration capabilities and a tendency to converge prematurely to local optima, which can decrease its performance in complex scenarios. This paper presents a modified form of the previously proposed AO, the Locality Opposition-Based Learning Aquila Optimizer (LOBLAO), aimed at resolving such issues and improving the performance of tasks related to global optimization and data clustering in particular. The proposed LOBLAO incorporates two key advancements: the Opposition-Based Learning (OBL) strategy, which enhances solution diversity and balances exploration and exploitation, and the Mutation Search Strategy (MSS), which mitigates the risk of local optima and ensures robust exploration of the search space. Comprehensive experiments on benchmark test functions and data clustering problems demonstrate the efficacy of LOBLAO. The results reveal that LOBLAO outperforms the original AO and several state-of-the-art optimization algorithms, showcasing superior performance in tackling high-dimensional datasets. In particular, LOBLAO achieved the best average ranking of 1.625 across multiple clustering problems, underscoring its robustness and versatility. These findings highlight the significant potential of LOBLAO to solve diverse and challenging optimization problems, establishing it as a valuable tool for researchers and practitioners.

## Introduction

In recent decades, different modified and improved metaheuristic optimization algorithms have been developed to provide higher-quality solutions for optimization problems, including global optimization^1,2^. The main idea is to enhance the searchability of a metaheuristics algorithm by modifying its operators using an efficient technique that can boost solution quality and improve the acceleration of the convergence speed. There are various applications for the metaheuristics optimization algorithms, for example, enhancing time-series forecasting^3^, solving global optimization problems^1^, manufacturer machine scheduling^4^, feature selection^5,6^, data clustering^7^ and many other applications^1,2^.

For example, the differential evolution was applied in^8^ to solve unconstrained global optimization problems. The differential evolution was improved using four crossover operators; called binomial, simple arithmetic, uniform arithmetic, and single arithmetic crossover. The four operators were employed to improve its performance and to avoid the problem of the original crossover operators of the differential evolution. It was evaluated with four standard benchmarking functions and achieved significant results. Houssein et al.^9^ developed an enhanced version of the marine predators’ algorithm. The opposition-based learning technique was adopted to boost the traditional MPA’s searchability and accelerate convergence speed. It was compared to several old and new metaheuristics methods modified by OBL, and the MPA-OBL obtained the best results in different datasets.

The artificial bee colony algorithm was also used to address the global optimization issue by Chu et al.^10^. They used adaptive heterogeneous competition to augment the ABC and boost its solution quality. It was compared to several ABC variants and other metaheuristics optimization methods and performed better than them in different benchmark function datasets. Two variants of the grey wolf optimizer were developed in^11^. The first one is called expanded Ex-GWO. The Ex-GWO has the same main three wolves of the traditional GWO: delta, alpha, and beta. Depending on the first three wolves, the next wolves update their positions in each iteration. The second one, incremental I-GWO, depends on the incremental model. The main idea of the I-GWO is that each wolf updates its position according to the wolves selected before it. Ex-GWO and I-GWO were evaluated on 33 benchmark functions and showed good performance. Cuong-Le et al.^12^ developed a novel version of GWO, namely NB-GWO, based on a balance function. NB-GWO was utilized to optimize hyperparameters of deep neural networks.

Zhang et al.^13^ developed an enhanced salp swarm algorithm (SSA) for solving global optimization problems using different strategies, such as generalized oppositional learning, quadratic interpolation, and orthogonal learning. The developed ESSA was evaluated in well-known benchmark functions with extensive comparisons to the traditional SSA and other optimizers and achieved significant results. Additionally, there are various modified metaheuristics algorithms, such as an enhanced sine cosine algorithm^14^, the modified whale optimization algorithm^15^, Quantum-inspired differential evolution^16^, a memory-based optimization algorithm^17^, a hybrid of butterfly and flower pollination optimization algorithm^18^ and an enhanced manta ray foraging optimizer^19^.

Metaheuristic algorithms are widely used these days for solving intricate optimization problems for the reason that they can be derived from a variety of nature and artificial phenomena. Many researchers have tried to understand the base and efficacy of these algorithms and studied their structures, performance, and versatility with respect to different optimization problems. Important developments and understanding have also been achieved in methods known as Grey Wolf, Moth Flame, Whale, Firefly, Bat, and Antlion Optimizations. Moreover, research on algorithms such as Whale Optimization Algorithm (WOA), and Chimp Optimization Algorithm (ChOA) have looked into their operational working and benchmarks for performance metrics and open improvement areas^20–22^. These studies are aimed at revealing the foundations of metaheuristics further to sharpen a more vigorous approach towards the optimization problem.

Like global optimization, metaheuristics methods have also been adopted to solve data clustering problems depending on the hybridization concept. For example, Han et al.^23^ developed a new gravitational search algorithm variant to address the data clustering issue. The modified variant is called bird flock GSA, where a new mechanism inspired by birds’ collective response behavior was developed to add diversity to the traditional GSA. The developed BFGSA was compared to several basic optimization methods, including the conventional GSA, and showed superior performance. A hybrid Harris Hawks optimization algorithm with differential evolution was developed by^24^ for data clustering. The operators of the differential evolution were utilized to boost the exploitation process (local search) of the traditional HHO. This hybrid model showed significant performance compared to the conventional differential evolution and HHO. In^25^, a modified genetic algorithm, called a multi-objective GA, was proposed to be employed with fuzzy c-means for solving data clustering. Kaur et al.^26^ proposed an efficient clustering approach using a chaos and flower pollination algorithm hybrid over k-means. The chaotic FPA was compared to several well-known optimizers, including the original FPA, which performed better. There are also various boosted metaheuristics algorithms developed for data clustering, such as an improved Black-Hole algorithm^27^, moth-flame optimization algorithm^28^, an improved ABC using WOA^29^ and multi-verse optimizer^30^.

Metaheuristic algorithms are also referred to as optimization techniques as they are formed from inspiration drawn from natural processes, biological systems, or physical phenomena. Some prominent examples of metaheuristic algorithms include genetic algorithms, particle swarm optimization, ant colony optimization, and differential evolution. There are myriad applications of these algorithms in almost every domain, such as engineering design, machine learning, etc. This is due to their inherent capacity to do two things optimally: global search across the entire solution space and local search in scope in order to refine the solutions for optima. However, no algorithm is free of shortcomings; for instance, some studies suggest that GA suffers from slow convergence. PSO and ACO are prone to becoming stuck in local optima in high-dimensional spaces. These challenges provide the impetus for the development of new algorithms of metaheuristic properties that can target one or more optimization objectives. In The last few years, many cutting-edge algorithms have emerged specifically to tackle these shortcomings found in the traditional metaheuristic approaches. So far, metaheuristic algorithms like Grey wolf optimizer, whale optimization algorithm, and slime mold algorithm have proven to be effective in processing high dimensional and complex optimization problems. These algorithms also adapt new mechanisms like novel strategies for searching the solution space, better balancing between exploration and exploitation, and others, which result in better and faster algorithms. Nevertheless, there remain many challenges that are yet to be solved; issues like scalability, multiple other scenarios in a variety of problem landscapes, and efficiency emerge as ones requiring attention. In this scenario, Aquila Optimizer has been invented as an add-on, but this tool still has a performance gap, specifically in high dimensional optimization; thus, LOBLAO is proposed.

The clustering problem is a ubiquitous challenge across various domains, where the goal is to group similar data points while maintaining separation between different groups. This problem arises in machine learning, data analysis, and pattern recognition. However, due to real-world data’s complexity and feature spaces’ high dimensionality, traditional methods often struggle to find accurate and efficient solutions. Addressing the clustering problem demands sophisticated optimization techniques that can handle large datasets, high-dimensional spaces, and non-convex relationships^31,32^. These optimization methods play a pivotal role in unraveling underlying patterns within data and are crucial for achieving meaningful insights and decision-making in complex scenarios.

In this paper, we introduce a new clustering method based on metaheuristics, utilizing an enhanced version of the Aquila Optimizer (AO) algorithm, which we call the Locality Opposition-based Learning Aquila Optimizer (LOBLAO). The AO algorithm is a recently developed optimization technique that mimics the natural behaviors of Aquila^33^. Its original implementation demonstrated strong search capabilities, performing effectively across various optimization tasks and surpassing several earlier metaheuristic algorithms^33^. However, like many metaheuristic methods, AO has certain limitations in its search capabilities. In order to tackle these problems, we incorporate the Opposition-Based Learning (OBL) technique into traditional AO to extend the population diversity and collaboration of the search strategies. Furthermore, to deal with the problem of local searches focusing on the same areas, we strengthen the exploration of new search areas by using a Mutation Search Strategy (MSS). The LOBLAO method is verified in the context of global optimization and data clustering problems. The effectiveness of the LOBLAO algorithm was compared with both standard AO and various other metaheuristic optimization algorithms on a set of well-known benchmark functions.

In short, this study proposes the following contribution:

- A new Aquila Optimizer algorithm variant, called LOBLAO, is proposed to solve global optimization and data clustering problems.
- Enhancing solution quality of the traditional AO algorithm using the Opposition-based Learning (OBL) technique. The OBL can keep the diversity of the solutions and maintain the equilibrium between the search mechanisms.
- Enhancing the search process of the traditional AO using the Mutation Search Strategy (MSS), which can be used to find new search regions.
- Implement extensive comparisons to verify the performance of the developed LOBLAO in both global optimization and data clustering problems.

To sum up, the structure of the current study is as follows. Section “Background and algorithms” presents the basic methods used in the main steps of the proposed method. Section “The developed approach (LOBLAO)” highlights the design of the developed LOBLAO and shows the main operators used and their modifications. Section “Experiments and results” illustrates the global optimization and data clustering evaluation experiments and their settings. Section “Conclusion and future works” concludes the current study and proposes a set of future work directions.

## Background and algorithms

### Data clustering problem

Based on the common property of having a *d*-dimensional range, the data clustering problem involves dividing *N* data objects into multiple clusters, denoted as *K* (groups). A set of *N* data elements in the *d*-dimensional space can be represented as $C = \left\{ c_1, c_2, c_3,\ldots , c_n \right\}$. Conversely, the *k* groups can be expressed as $X = \left\{ x_1, x_2, x_3,\ldots , x_n \right\}$. Typically, each group should contain at least one member or data item.

1 $$\begin{aligned} X_{i} \ne \phi , \forall \in \left\{ 1,2,\ldots ,k \right\} \end{aligned}$$

At the same time, no more than two clusters should share a data object or a member.

2 $$\begin{aligned} X_i\cap Xj = \phi , \forall i \ne j \, and \, i,j \in {1,2,\ldots ,k} \end{aligned}$$

A group should be assigned to each data object.

3 $$\begin{aligned} C = \bigcup _{i=1} ^{k} X_i \end{aligned}$$

For information consolidation purposes, the distance test is important. Often, the shortest distance between two data sets is the distance between two points in that space defined by characteristic features of the two data sets. For this purpose, the most known measurement is the Euclidean distance. At the same time, the general Euclidean separation is used in the calculations of clustering to estimate the quality of the clusters^24^.

The Euclidean distance (*E*.*d*) between the data points *p*, and *q* can be calculated.

4 $$\begin{aligned} E.d (D_p, D_q) = \sqrt{\sum _{s=1}^{l} (D_{p,s} - D_{q,t})^2 } \end{aligned}$$

where *q* represents the $\hbox {q}_{th}$ data point, and *l* indicates the length of the dimension. Additionally, the total intra-cluster distance is the most frequently utilized metric^34^. The whole intra-cluster remove degree determines the approximate separation between data points of any cluster and the barycenter of that cluster.

5 $$\begin{aligned} Intra _{sum}= \sum _{q=i}^k \left\| X_{cq} - C_{q} \right\| \end{aligned}$$

where *q* is the $\hbox {q}_th$ data point, and $\hbox {C}_q$ is given to the $\hbox {q}_{th}$ cluster’s barycenter.

### Aquila optimizer (AO)

This section provides a basic overview of the Aquila Optimizer (AO). The AO algorithm, introduced by^33^, mimics the social behavior of Aquila when hunting for prey in the wild. The AO is classified as a population-based optimization method, starting with the generation of an initial population *X* that has *N* dimensions. This process is described by Eq. (6).

6 $$\begin{aligned} X_{ij}=r_1 \times (UB_j-LB_j)+LB_j,\, \,\, j=1,2,3,\ldots ,Dim, \, \, i=1,2,3,\ldots ,N \end{aligned}$$

The search domain’s limitations are $UB_j$ and $LB_j$. *Dim* is the population’s dimension. $r_1 \in [0,1]$ is the random value.

The next steps are to explore or exploit the search space, and they are repeated till founding the best solution. According to^33^, the AO uses two methods for applying the exploration and exploitation.

The agent’s best ($X_b$) and the average ($X_M$) are used in the first strategy to perform the exploration as in Eqs. (7–8).

7 $$\begin{aligned} & X_i(t+1)=X_b(t)\times \left( \frac{1-t}{T} \right) + ( X_{M}(t) -X_b(t) *rand), \end{aligned}$$

8 $$\begin{aligned} & X_{M}(t)=\frac{1}{N}\sum _{i=1}^{N}X(t) \end{aligned}$$

Here, $\left( \frac{1-t}{T} \right)$ keeps the search active during the exploration phase. *T* represents the maximum number of iterations.

The second technique, formulated as follows, employs the Levy flight (*Levy*(*D*) distribution and $X_b$ to update the solutions’ exploration capabilities.

9 $$\begin{aligned} & X_i(t+1)=X_b(t)\times Levy(D)+X_R(t)+(y-x)*rand, \end{aligned}$$

10 $$\begin{aligned} & Levy(D)=s \times \frac{u \times \sigma }{|\upsilon |^{\frac{1}{\beta }}}, \, \sigma =\left( \frac{\Gamma (1+\beta ) \times sine(\frac{\pi \beta }{2} )}{\Gamma (\frac{1+\beta }{2}) \times \beta \times 2^{(\frac{\beta -1}{2})}} \right) \end{aligned}$$

where $\upsilon$ and *u* denote random numbers. $beta=1.5$ and $s=0.01$ are constants. $X_R$ is an agent selected randomly in Eq. (9). In addition, *x* and *y* are utilized to simulate the spiral shape as in the following equations:

11 $$\begin{aligned} & x= sin(\theta ) \times r, \, \, \, \ y=cos(\theta ) \times r \end{aligned}$$

12 $$\begin{aligned} & r=r_1+U \times D_1, \, \theta = -\omega \times D_1 +\theta _1, \, \theta 1=\frac{\pi \times 3 }{2} \end{aligned}$$

here $U=0.00565$ and $\omega =0.005$. $r_1$ is selected randomly $\in [0,20]$.

The first way is used in^33^ to update agents in the exploitation stage using $X_M$ and $X_b$ as follows:

13 $$\begin{aligned} X_i(t+1)= ((UB-LB) \times rand+LB)\times \delta + ( X_b(t)-X_{M}(t) )\times \alpha - rand \end{aligned}$$

The parameters for exploitation adjustment are denoted by $\delta$ and $\alpha$, with a random value (*rand*) ranging from [0, 1].

The agent’s update is influenced by the quality of the function (*QF*), *Levy*, and $X_b$ in the subsequent exploitation strategy. This process is defined as follows:

14 $$\begin{aligned} & X_i(t+1)= X_b(t) \times QF -(G_1\times X(t)\times rand)-G_2\times Levy(D)+ G_1 \times rand \end{aligned}$$

15 $$\begin{aligned} & QF(t)=t^{(\frac{rand \times 2 -1}{(1-T)^2})} \end{aligned}$$

**Figure 1:**
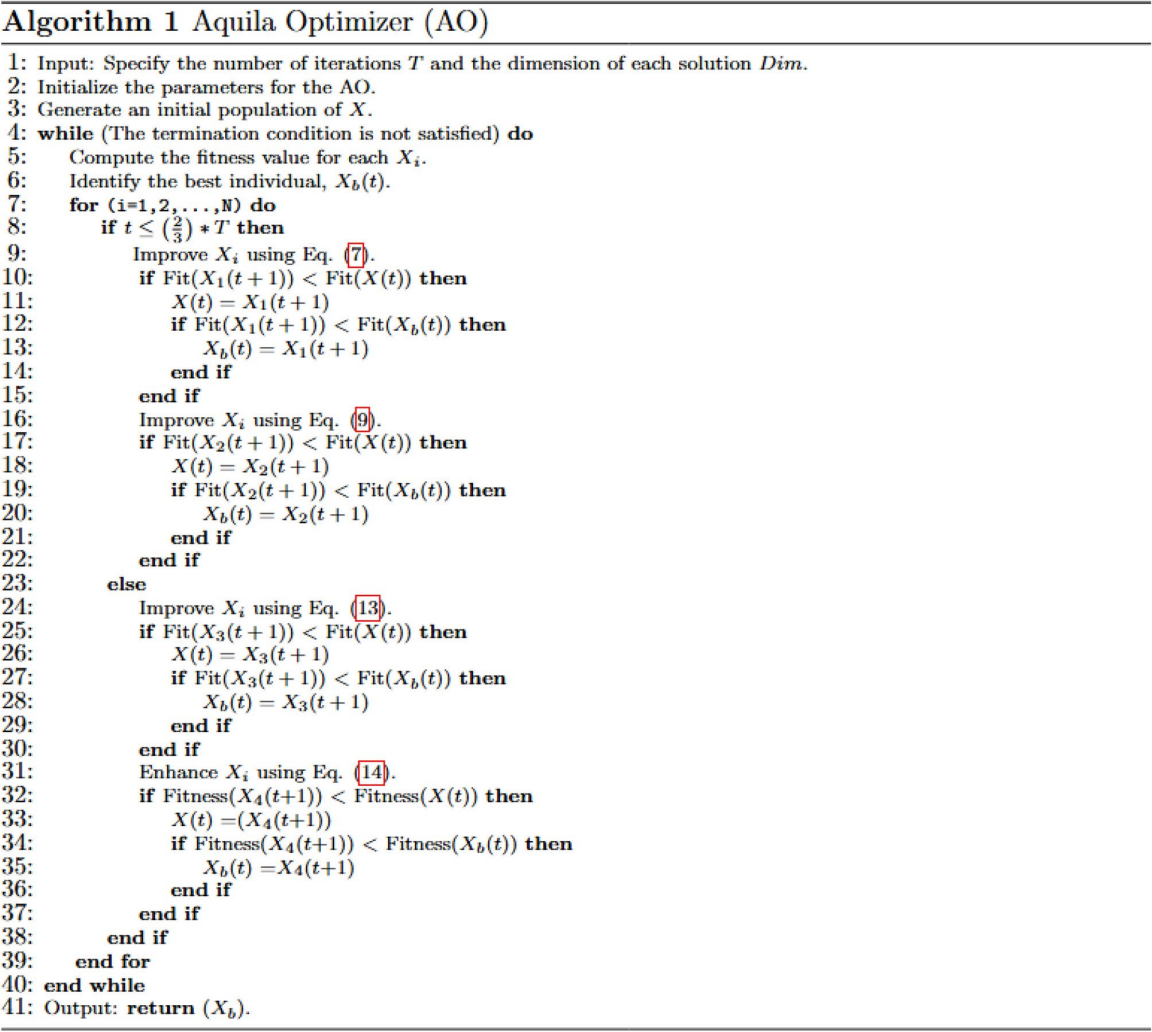
Aquila Optimizer (AO).

Furthermore, $G_1$ denotes several motions applied to find the optimal individual solution, as follows:

16 $$\begin{aligned} G_1=2 \times rand-1, \, \, \, G_2=(1-\frac{t}{T}) \times 2 \end{aligned}$$

where $G_2$ is used to decrease the values from 2 to 0. The exact steps of the AO method are shown in Fig. 1.

### Opposition-based learning

Opposition-based learning (OBL) is a machine intelligence approach^35^ that has been utilized to enhance the performance of various optimization techniques^36,37^. The OBL strategy focuses on generating an opposition solution, which aims to identify a better candidate solution that yields a superior fitness value and moves closer to the optimal solution.

The opposite value of ${\overline{X}}$ for a given value $X\in [UB,LB]$ is defined as:

17 $$\begin{aligned} {\overline{X}}=UB+LB-X \end{aligned}$$

Assuming ${\overline{X}}$ = ($X_1$, $X_2$, ..., $X_n$) represents a point in a multi-dimensional space, where $X_1$, $X_2$, ..., $X_D$ $\in$ *R* and $X_j$ is within [$UB_j$, $LB_j$], for *j* $\in$ 1, 2, ..., *D*. The definition of ${\overline{X}}$ in n dimensions can be expressed as:

18 $$\begin{aligned} \overline{X_j}=UB_j+LB_j-X_j, \quad \quad {\text {where}} \quad j=1\ldots D. \end{aligned}$$

Furthermore, the supplied two solutions (${\overline{X}}$and *X*) are compared according to their fitness functions in the optimization phase, with the best solution being stored and the other being discarded. If *f*(*X*) *leq* *f*(${\overline{X}}$), *X* is saved in the minimization case; otherwise, ${\overline{X}}$is saved.

### Mutation search strategy (MSS)

In genetic algorithms, the mutation operator is very significant. By creating a uniformly distributed random value between [0, 1],^38^, the mutation probability *Mu* is used as a control parameter for tuning the mutation operator. The mutation operator is defined as follows:

19 $$\begin{aligned} X_{ij}= {\left\{ \begin{array}{ll} Xb_j+\mu (X_{pj}-X_{qj}) & if \, rand()>\mu _r\\ X_{ij}& Otherwise \end{array}\right. } \end{aligned}$$

where $$\mu _r = 0.5$$, $$p, q \in \{1, 2,\ldots ,i - 1, i + 1,\ldots , N\}$$ and $$\mu \in [0,1]$$.

## The developed approach (LOBLAO)

This section describes the so-called Locality Opposition-based Learning Aquila Optimizer (LOBLAO), which is a new approach developed to increase the robustness of the basic Aquila Optimizer (AO) in relation to high dimensional and complex optimization problems. The technique contains three principal strategies-Aquila Optimizer Operators, Opposition Based Learning (OBL), and Mutation Search Strategy (MSS)-that interact with each other such that the identified limitations of the original AO are obviated. The incorporation of these strategies leads to LOBLAO achieving an improved balance between exploration and exploitation, enhancement of solution diversity as well and alleviation of early convergence to a solution.

### Strategies for improvement in LOBLAO

**Figure 2:**
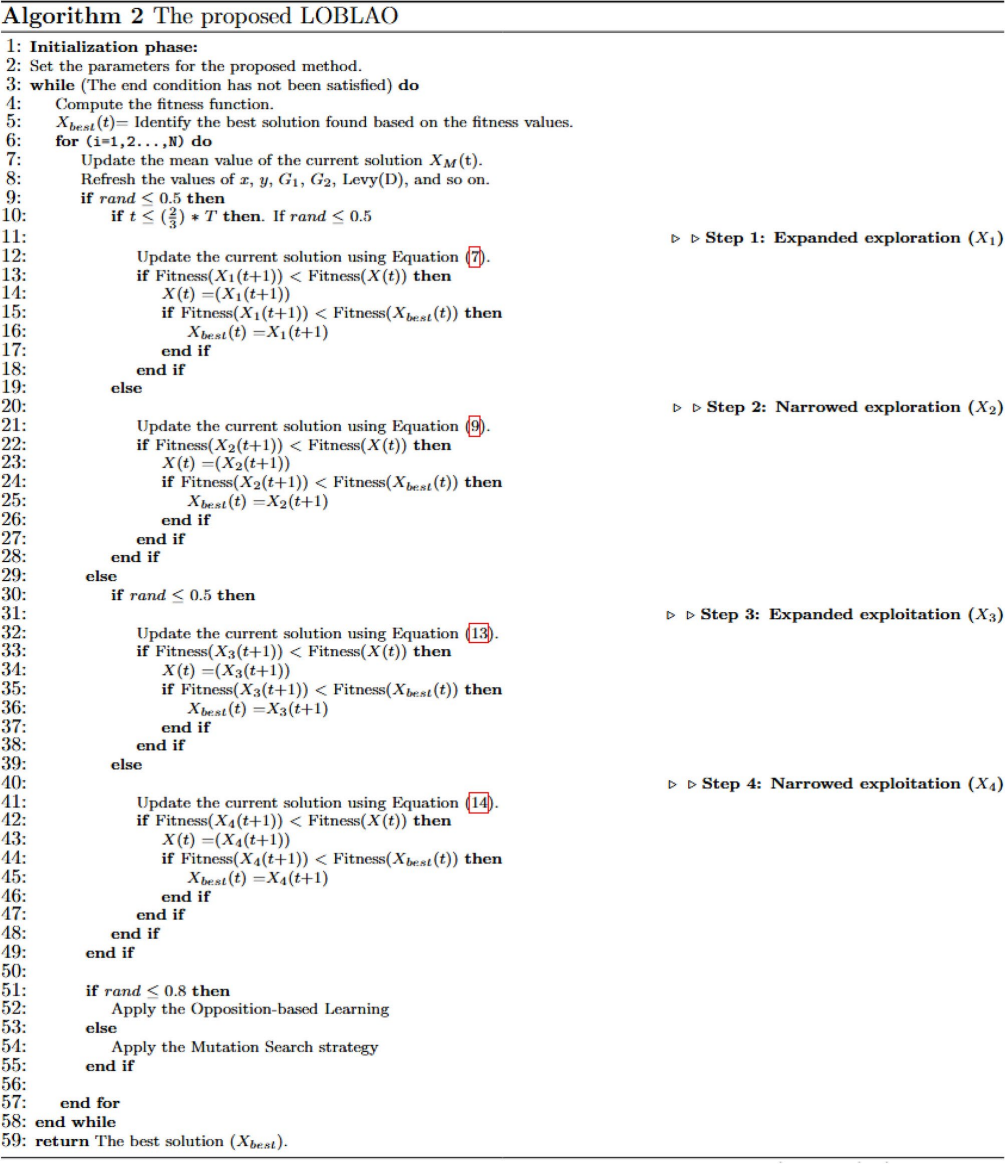
The proposed LOBLAO.

The proposed LOBLAO in Fig. 2 uses three additional methods to mitigate the weaknesses of the original AO:

#### Aquila optimizer operator

The core components of Aquila Optimiser are still retained, which are the building blocks of LOBLAO, and this is necessary since these components form a suitable and effective framework of modeling, exploration, and exploitation based firmly on the hunting and flight movement of the Aquila bird. These operators also assist in scaling the searching of strategies and in maintaining that LOBLAO retains notable features as AO, which includes being adaptable and simple.

#### Opposition-based learning (OBL) strategy

The strategy helps to increase the diversity of the solutions by using both the current and the current opposite solution. Instead of following the conventional OBL methods, the OBLAO model uses a *least OBL* approach, which creates oppositional solutions within a limited boundary of the search space. This centripetal technique aids in eliminating the unfocused and unwarranted movement in other areas while still retaining diversity. With the addition of localized OBLs, LOBLAO is able to effectively advance and make sure that the algorithm is always active and self-adjusting to a myriad of problem topologies.

#### Mutation search strategy (MSS)

In the way that LOBLAO is able to locate high dimensional local maxima, MSS has been able to due to it being an inclusion strategy. The Modification Search Strategy takes controlled variations of possible solutions, leading to the exploration of new regions that have not been explored before around the solution space. Combined with robust search patterns and other measures like MSS, the modification search strategy achieves tremendous effectiveness in overcoming the challenge of early convergence. MSS’s inclusion, along with robust search patterns, ensures that LOBLAO will not be trapped in local optimal solutions and will constantly progress its searching direction over complex multi-dimensional spaces.

### Workflow of LOBLAO

Figure 2 outlines the specifications of the newly LOBLAO procedure. In every iteration of the model, three strategies are applied: Aquila Optimizer operators, OBL, or MSS. In every iteration, given probabilities are randomly assigned to these three strategies, which are biased towards one of them, leading to a certain strategy being selected and applied. In this manner, the algorithms ensure that the contribution of all the strategies in a team-based optimization task is equal.

1. Initialization: The algorithm generates a random initial candidate population throughout the search space.

2. Fitness Evaluation: The second step involves measuring the fitness value for every candidate solution through the objective function.

3. Search Strategy Selection: One of the three strategies (AO, OBL, or MSS) is implemented by the algorithm in every iteration to perform updates to the candidate solutions

4. Updating Population: The population is updated according to the selected strategy, and the best solutions are kept for the next iteration.

5. Termination: This final stage starts when the maximum number of evaluations of the fitness or iterations has been reached.

### Advantages of LOBLAO

New enhancements are done on LOBLAO so that it can work on the concurrent limitations of the original AO as well as other modern algorithms:

- Enhanced Exploration and Exploitation: The combination of the AO operators and OBL and MSS is good for LOBLAO as it helps achieve a good balance between global and local search ranges.
- Improved Solution Diversity: Applying a localized OBL allows the search process to target a wide range of potential solutions and, therefore, improves the diversity of the solution set.
- Resilience to Local Optima: With the help of MSS, which involves randomness and variability, the algorithm is able to break away from local optima and look for other areas.
- Efficiency in High-Dimensional Problems: The specific and directed approach of LOBLAO’s strategies ensures that it can be employed efficiently in optimization tasks that are complex and high-dimensional, and the scaling is also reasonable.

The modifications incorporated in LOBLAO make it an efficient optimization tool that can address a wide range of complex problems. This section illustrates the workings and efficiency of the proposed method using detailed experiments with benchmark test functions and data clustering problems.

### Computational complexity of the proposed LOBLAO

The recommended computational complexity of LOBLAO is influenced by how candidate solutions are initialized, how the objective function of the current solutions is evaluated, and how candidate solutions are updated iteratively.

Let’s assume that *N* represents the total number of employed solutions, and *O*(*N*) indicates the time complexity for initializing these solutions. The time complexity for updating the solutions can be expressed as *O*(*T* $\times$ *N*) + *O*(*T* $\times$ *N* $\times$ *Dim*), where *T* is the total number of iterations and *Dim* refers to the spatial dimension of the problem. Therefore, the time complexity for the LOBLAO can be described as follows.

20 $$\begin{aligned} O(LOBLAO)=(N)\times O(AO)+ O(OBL) + O(MSS) \end{aligned}$$

The time complexity of the method under consideration is contingent on the interplay of three primary search operators: AO, OBL, and MSS. The computation of the time complexity for these methods is delineated below.

21 $$\begin{aligned} & O(OBL)=O(N\times (T\times Dim+1)) \end{aligned}$$

22 $$\begin{aligned} & O(AO)=O(N\times (T\times Dim+1)) \end{aligned}$$

23 $$\begin{aligned} & O(MSS)=O(N\times Dim) \end{aligned}$$

Henceforth, the comprehensive time complexity of LOBLAO can be expressed as follows.

24 $$\begin{aligned} & O(LOBLAO)=O(T \times N\times (Dim+1) +( N\times Dim) +( N\times Dim)) \end{aligned}$$

25 $$\begin{aligned} & O(LOBLAO)=O\left( T \times N \times \left( Dim+N\right) \right) \end{aligned}$$

## Experiments and results

This section outlines the experiments conducted and the results obtained from both the proposed methods and other comparative approaches. Two experiments were carried out: the first focused on global optimization, while the second addressed clustering problems. All experiments utilized MATLAB R2015a, running on an Intel(R) Core(TM) i7 processor with 16GB of RAM. The global parameters were configured with a population size of 30 and a total of 500 iterations. To ensure statistical validity, each experiment was executed with 30 independent runs.

### Experiments 1: Benchmark functions problems

This experiment assesses the performance of the LOBLAO by utilizing 23 established benchmark functions^39^ and 30 CEC 2017 benchmark functions^40^. The LOBLAO was executed for 500 iterations with 30 candidate solutions to tackle these test functions. To evaluate the reliability of the LOBLAO, the algorithm was run independently 30 times; we documented the best function value, average outcomes, worst function value, standard deviation (STD), p-value, and rank. The subsequent sections will compare the proposed algorithm with 11 prominent state-of-the-art algorithms. To ensure a fair comparison, all algorithms were set to the same population size and iteration counts of 30 and 500, respectively.

### Description of benchmark functions

**Figure 3:**
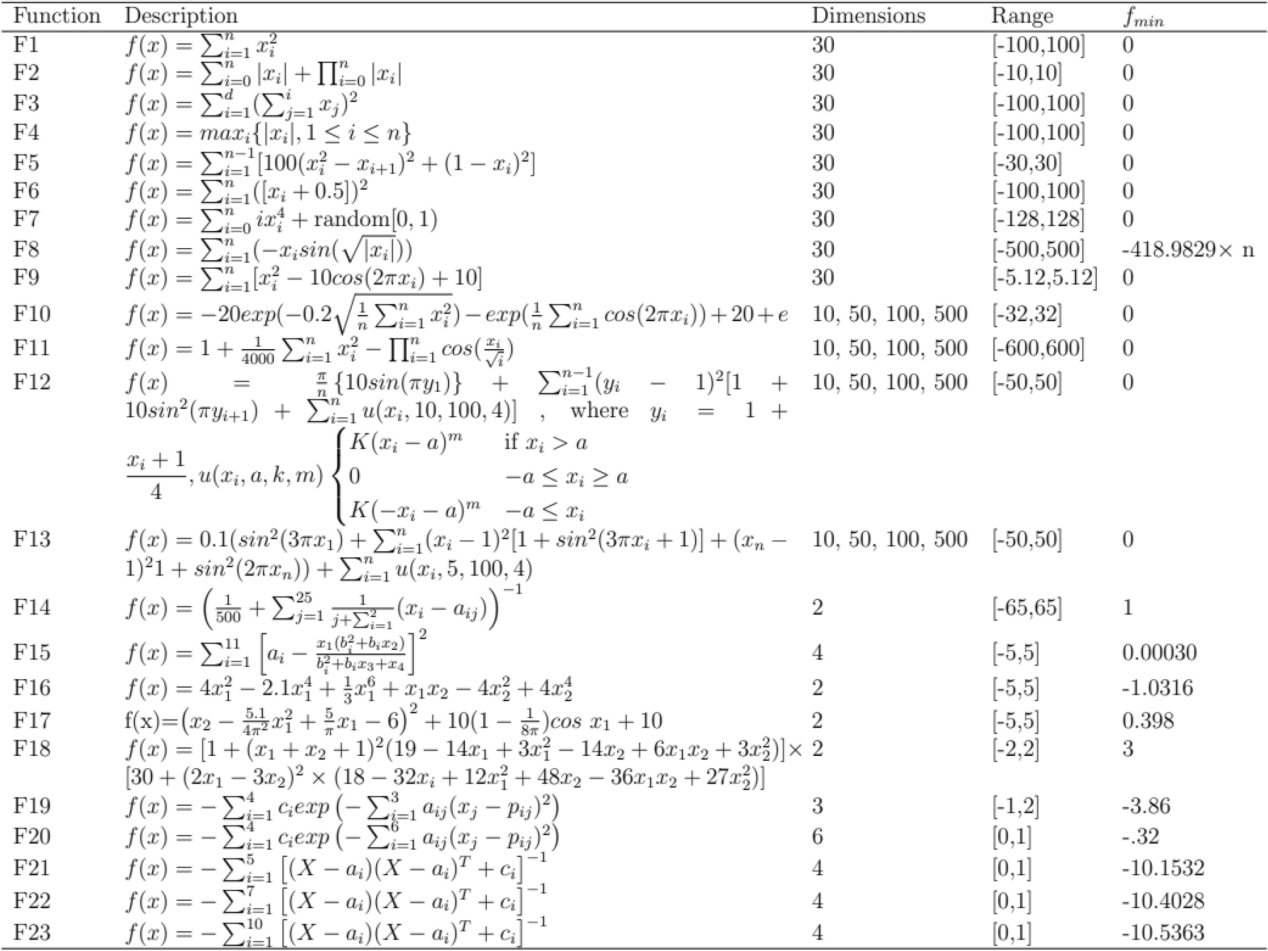
Details of the tested benchmark functions.

To evaluate the exploratory and exploitative behaviors of the LOBLAO, we utilized 23 benchmark functions that encompass various problem types: multimodal, fixed-dimension multimodal, and unimodal functions^39^. The LOBLAO will be tested on unimodal functions (F1–F7) (refer to Fig. 3) to assess its exploitation tendencies. Additionally, the multimodal benchmark functions (F8–F13) will be employed to evaluate the exploration capabilities of the LOBLAO. Both 10 and 100 dimensions are used in these two groups of functions. To further investigate the exploration ability of the LOBLAO in lower dimensions, we will use the fixed-dimension multimodal functions (F14–F23) (see Table 3). A variety of well-known algorithms are compared with the proposed algorithm to demonstrate the superior performance of the LOBLAO. The parameter values for the comparative algorithms are detailed in Table 1. The code was developed using the MATLAB R2015a platform and executed on a PC equipped with 16 GB RAM and an Intel(R) Core(TM) i7 CPU.

**Table 1:** Parameters values of the tested algorithms.

No.	Algorithm	Parameter	Value
1	AOA	$\alpha$	5
		$\mu$	0.5
2	SSA	$v_0$	0
3	WOA	$\alpha$	Decreased from 2 to 0
		*b*	2
4	SCA	$\alpha$	0.05
5	DA	*w*	0.2–0.9
		*a*, *s*, and *c*	0.1
		*e* and *f*	1
6	GWO	*a*	Linear reduction from 2 to 0
7	PSO	Topology	Fully connected
		Cognitive and social constant	(C1, C2) 2, 2
		Inertia weight	Linear reduction from 0.9 to 0.1
		Velocity limit	10% of dimension range
8	ALO	*I* ratio	$10^w$
		*w*	2–6
9	MPA	$\gamma$	$\gamma$>1
		*P*	0.0
10	EO	*r*	0.5
		*a*	4
		*GP*	0.5
1	AO	$\alpha$	0.1
		$\delta$	0.1

#### Analysis of LOBLAO convergence

**Figure 4:**
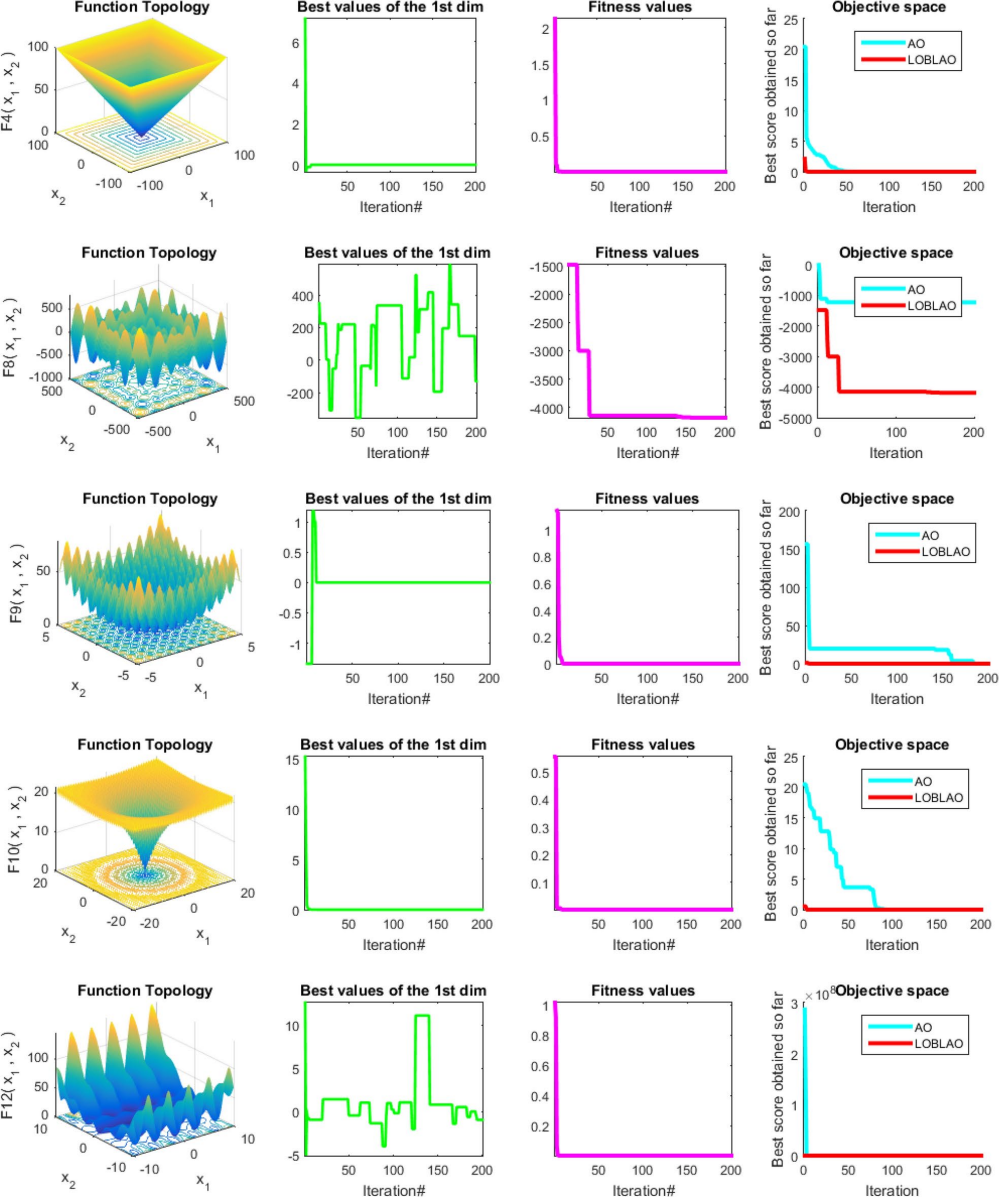
Qualitative results for the studied problems.

To illustrate the behavior of the LOBLAO algorithm, we plot the trajectory and convergence curves in Fig. 4. This figure presents qualitative measures, including the 2D function topology (first column), the best values of the first dimension (second column), the average fitness value of the LOBLAO algorithm (third column), and the convergence curves for both the AO and LOBLAO algorithms (fourth column). In the second column, the curve for the best values of the first dimension indicates that the solution begins with a high frequency and magnitude. However, in the last iterations, these values become obscured in functions F4, F9, and F10. This suggests that LOBLAO demonstrates strong exploration capabilities initially, followed by effective exploitation towards the end. This pattern indicates that the proposed algorithm has a significant chance of reaching the optimum. The third column shows the average fitness value of all candidate solutions over the iterations. It is observed that the average fitness value is initially high. Still, it decreases before the 40th iteration, suggesting that the LOBLAO algorithm requires only a few iterations to find the optimum. In the fourth column, the convergence curves for the AO and LOBLAO algorithms indicate that LOBLAO converges more quickly than AO. In some cases, such as with function F8, LOBLAO achieves a significantly better solution than AO. Additionally, the convergence curves are smooth, and LOBLAO improves with just a few iterations.

#### Parameter analysis of the LOBLAO algorithm

In this part, Table 2 shows the effect of changing the solution number (N) of the LOBLAO on its behavior. To make a comprehensive analysis, several solution numbers are used (i.e., 5, 10, 15, 20, 25, 30, 35, 40, 45, and 50) to see the impact of this parameter throughout 500 iterations. Table 2 shows that the proposed algorithm keeps its power and robustness. From the rank in Table 2, one can see that the higher the solutions number (*N*) is, the higher the performance is. The LOBLAO with ($N=50$) has the first or second ranks in 11 functions, except for F7 and F13. Table 2 shows that the change in the solutions number in the functions F9, F10, and F11 does not affect the performance of LOBLAO. One can conclude that the best solution number is 45 because the LOBLAO with ($N=45$) has the first final rank. The Wilcoxon signed-rank test ($\alpha = 0.05$) is performed between the LOBLAO and the other cases ($N=50$). The p-value and *h* are provided. For a certain *N*, its h equals zero means that the LOBLAO with ($N=50$) is statistically different from this *N* value. Otherwise, the LOBLAO with ($N=50$) is statistically different from this *N* value.

**Table 2:** The effect of the number of solutions (*N*) on the performance of the proposed method.

Fun	Measure	Number of solutions									
		5	10	15	20	25	30	35	40	45	50
F1	Best	3.0098E−198	1.0272E−259	0.0000E+00	0.0000E+00	0.0000E+00	0.0000E+00	0.0000E+00	0.0000E+00	0.0000E+00	0.0000E+00
	Average	7.5245E−199	2.5679E−260	0.0000E+00	0.0000E+00	0.0000E+00	0.0000E+00	0.0000E+00	0.0000E+00	0.0000E+00	0.0000E+00
	Worst	8.5198E−229	6.4198E−260	0.0000E+00	0.0000E+00	0.0000E+00	0.0000E+00	0.0000E+00	0.0000E+00	0.0000E+00	0.0000E+00
	STD	0.0000E+00	0.0000E+00	0.0000E+00	0.0000E+00	0.0000E+00	0.0000E+00	0.0000E+00	0.0000E+00	0.0000E+00	0.0000E+00
	p-value	0.0000E+00	0.0000E+00	NaN	NaN	NaN	NaN	NaN	NaN	NaN	NaN
	h	1	1	NaN	NaN	NaN	NaN	NaN	NaN	NaN	NaN
	Rank	10	9	1	1	1	1	1	1	1	1
F2	Best	1.6418E−96	1.6715E−125	9.2811E−154	2.0435E−191	1.3310E−220	1.8357E−217	1.2724E−231	1.5975E−237	1.1549E−262	3.8185E−260
	Average	4.1044E−97	4.1787E−126	2.3203E−154	5.1087E−192	3.3274E−221	4.5901E−218	3.1809E−232	3.9938E−238	2.8993E−263	9.5463E−261
	Worst	8.8154E−110	9.7287E−152	2.1545E−191	1.3224E−210	3.8043E−230	7.5668E−241	1.6890E−258	1.9472E−274	1.9052E−275	8.4715E−286
	STD	8.2088E−97	8.3575E−126	4.6406E−154	0.0000E+00	0.0000E+00	0.0000E+00	0.0000E+00	0.0000E+00	0.0000E+00	0.0000E+00
	p-value	3.5592E−01	3.5592E−01	3.5592E−01	0.0000E+00	0.0000E+00	0.0000E+00	0.0000E+00	0.0000E+00	0.0000E+00	NaN
	h	0	0	0	1	1	1	1	1	1	NaN
	Rank	10	9	8	7	5	6	4	3	1	2
F3	Best	4.9051E−191	8.6604E−253	0.0000E+00	0.0000E+00	0.0000E+00	0.0000E+00	0.0000E+00	0.0000E+00	0.0000E+00	0.0000E+00
	Average	1.2263E−191	2.1651E−253	0.0000E+00	0.0000E+00	0.0000E+00	0.0000E+00	0.0000E+00	0.0000E+00	0.0000E+00	0.0000E+00
	Worst	9.3881E−219	8.6719E−304	0.0000E+00	0.0000E+00	0.0000E+00	0.0000E+00	0.0000E+00	0.0000E+00	0.0000E+00	0.0000E+00
	STD	0.0000E+00	0.0000E+00	0.0000E+00	0.0000E+00	0.0000E+00	0.0000E+00	0.0000E+00	0.0000E+00	0.0000E+00	0.0000E+00
	p-value	0.0000E+00	0.0000E+00	0.0000E+00	NaN	NaN	NaN	NaN	NaN	NaN	NaN
	h	1	1	1	NaN	NaN	NaN	NaN	NaN	NaN	NaN
	Rank	10	9	1	1	1	1	1	1	1	1
F4	Best	7.4352E−100	1.1487E−125	4.0884E−153	2.0820E−191	2.4477E−221	3.4455E−220	5.7340E−230	3.1503E−238	8.9182E−264	7.2978E−262
	Average	1.8593E−100	2.8716E−126	1.0221E−153	5.2050E−192	6.1618E−222	8.6136E−221	1.4335E−230	7.8758E−239	2.2343E−264	3.0317E−262
	Worst	6.0264E−110	1.7484E−151	4.5214E−192	7.2472E−210	6.0117E−229	7.4347E−241	7.4799E−260	3.5445E−269	1.8137E−278	1.7396E−290
	STD	3.7173E−100	5.7433E−126	2.0442E−153	0.0000E+00	0.0000E+00	0.0000E+00	0.0000E+00	0.0000E+00	0.0000E+00	0.0000E+00
	p-value	3.5576E−01	3.5592E−01	3.5592E−01	0.0000E+00	0.0000E+00	0.0000E+00	0.0000E+00	0.0000E+00	0.0000E+00	NaN
	h	0	0	0	1	1	1	1	1	1	NaN
	Rank	10	9	8	7	5	6	4	3	1	2
F5	Best	4.5504E+00	5.6185E−01	1.6850E−01	6.9070E−02	1.0296E−01	1.7051E−01	6.6203E−03	1.4299E−02	1.3570E−03	5.6053E−03
	Average	1.6029E+00	2.5744E−01	4.5496E−02	2.7800E−02	2.7063E−02	5.0724E−02	2.0624E−03	5.0828E−03	4.5257E−04	1.8316E−03
	Worst	5.9491E−05	3.4662E−04	4.6390E−04	1.8325E−04	1.2663E−04	1.2305E−04	2.2363E−04	1.0830E−03	1.3118E−05	2.1556E−04
	STD	2.0668E+00	2.9917E−01	8.2054E−02	3.1550E−02	5.0637E−02	8.0766E−02	3.0470E−03	6.1796E−03	6.2349E−04	2.5479E−03
	p-value	1.7227E−01	1.3836E−01	3.2835E−01	1.5195E−01	3.5802E−01	2.7174E−01	9.1125E−01	3.6822E−01	3.3355E−01	NaN
	h	0	0	0	0	0	0	0	0	0	NaN
	Rank	10	9	7	6	5	8	3	4	1	2
F6	Best	4.7010E−02	2.2755E−02	2.1093E−04	2.5611E−04	5.7929E−04	1.0626E−04	1.3593E−05	8.2942E−06	8.1476E−05	1.4619E−05
	Average	1.1850E−02	5.7374E−03	7.3073E−05	1.0370E−04	1.4551E−04	3.2939E−05	6.3037E−06	4.3493E−06	2.8501E−05	6.0004E−06
	Worst	3.2322E−05	1.5416E−05	1.2279E−06	8.7792E−07	4.8415E−08	4.5138E−07	5.7926E−09	3.2289E−07	2.5145E−06	9.7651E−08
	STD	2.3440E−02	1.1345E−02	9.6569E−05	1.2416E−04	2.8918E−04	5.0143E−05	7.2742E−06	3.6110E−06	3.6587E−05	7.0761E−06
	p-value	3.5121E−01	3.5132E−01	2.1524E−01	1.6721E−01	3.7200E−01	3.2830E−01	9.5429E−01	6.9211E−01	2.7264E−01	NaN
	h	0	0	0	0	0	0	0	0	0	NaN
	Rank	10	9	6	7	8	5	3	1	4	2
F7	Best	1.3893E−03	3.6319E−04	5.8670E−04	8.2231E−04	3.1932E−04	2.4868E−04	2.7170E−04	1.0728E−04	8.0764E−05	3.2289E−04
	Average	5.9023E−04	2.2934E−04	3.2687E−04	2.8056E−04	1.4728E−04	7.7321E−05	1.3593E−04	6.7708E−05	4.3966E−05	1.0904E−04
	Worst	2.0468E−04	8.3975E−05	8.1466E−06	4.6794E−05	1.5554E−05	8.7111E−06	2.4433E−05	6.2225E−06	1.9141E−05	1.4128E−06
	STD	5.3959E−04	1.1736E−04	2.4133E−04	3.6599E−04	1.2619E−04	1.1459E−04	1.2911E−04	4.5822E−05	2.8289E−05	1.4505E−04
	p-value	1.3577E−01	2.4469E−01	1.7275E−01	4.1704E−01	7.0450E−01	7.4320E−01	7.9110E−01	6.0646E−01	4.1241E−01	NaN
	h	0	0	0	0	0	0	0	0	0	NaN
	Rank	10	7	9	8	6	3	5	2	1	4
F8	Best	−4.1714E+03	−4.1702E+03	−4.1744E+03	−4.1865E+03	−4.1684E+03	−4.1853E+03	−4.1866E+03	−4.1883E+03	−4.1891E+03	−4.1886E+03
	Average	−4.1779E+03	−4.1819E+03	−4.1817E+03	−4.1881E+03	−4.1835E+03	−4.1878E+03	−4.1887E+03	−4.1892E+03	−4.1894E+03	−4.1894E+03
	Worst	−4.1866E+03	−4.1898E+03	−4.1882E+03	−4.1896E+03	−4.1891E+03	−4.1897E+03	−4.1898E+03	−4.1898E+03	−4.1898E+03	−4.1898E+03
	STD	6.6631E+00	8.8443E+00	5.9963E+00	1.3678E+00	1.0077E+01	2.0153E+00	1.4337E+00	7.2367E−01	3.2975E−01	5.2857E−01
	p-value	1.3977E−02	1.4192E−01	4.3132E−02	1.4022E−01	2.8459E−01	1.8633E−01	4.0853E−01	7.5101E−01	9.6324E−01	NaN
	h	1	0	1	0	0	0	0	0	0	NaN
	Rank	10	8	9	5	7	6	4	3	2	1
F9	Best	0.0000E+00	0.0000E+00	0.0000E+00	0.0000E+00	0.0000E+00	0.0000E+00	0.0000E+00	0.0000E+00	0.0000E+00	0.0000E+00
	Average	0.0000E+00	0.0000E+00	0.0000E+00	0.0000E+00	0.0000E+00	0.0000E+00	0.0000E+00	0.0000E+00	0.0000E+00	0.0000E+00
	Worst	0.0000E+00	0.0000E+00	0.0000E+00	0.0000E+00	0.0000E+00	0.0000E+00	0.0000E+00	0.0000E+00	0.0000E+00	0.0000E+00
	STD	0.0000E+00	0.0000E+00	0.0000E+00	0.0000E+00	0.0000E+00	0.0000E+00	0.0000E+00	0.0000E+00	0.0000E+00	0.0000E+00
	p-value	NaN	NaN	NaN	NaN	NaN	NaN	NaN	NaN	NaN	NaN
	h	NaN	NaN	NaN	NaN	NaN	NaN	NaN	NaN	NaN	NaN
	Rank	1	1	1	1	1	1	1	1	1	1
F10	Best	8.8818E−16	8.8818E−16	8.8818E−16	8.8818E−16	8.8818E−16	8.8818E−16	8.8818E−16	8.8818E−16	8.8818E−16	8.8818E−16
	Average	8.8818E−16	8.8818E−16	8.8818E−16	8.8818E−16	8.8818E−16	8.8818E−16	8.8818E−16	8.8818E−16	8.8818E−16	8.8818E−16
	Worst	8.8818E−16	8.8818E−16	8.8818E−16	8.8818E−16	8.8818E−16	8.8818E−16	8.8818E−16	8.8818E−16	8.8818E−16	8.8818E−16
	STD	0.0000E+00	0.0000E+00	0.0000E+00	0.0000E+00	0.0000E+00	0.0000E+00	0.0000E+00	0.0000E+00	0.0000E+00	0.0000E+00
	p-value	NaN	NaN	NaN	NaN	NaN	NaN	NaN	NaN	NaN	NaN
	h	NaN	NaN	NaN	NaN	NaN	NaN	NaN	NaN	NaN	0
	Rank	1	1	1	1	1	1	1	1	1	1
F12	Best	2.4065E−03	2.7665E−03	1.9491E−04	4.8960E−04	4.8400E−04	7.2587E−05	8.9067E−05	1.3011E−05	4.0025E−05	1.9972E−05
	Average	1.1766E−03	1.1690E−03	5.5468E−05	1.4368E−04	1.2390E−04	2.9313E−05	2.2855E−05	7.7586E−06	1.1660E−05	9.9098E−06
	Worst	6.0746E−05	3.5159E−05	1.4610E−06	9.6291E−08	5.6365E−07	1.4597E−06	2.5035E−10	1.1457E−06	4.9773E−09	2.5739E−07
	STD	1.2738E−03	1.3404E−03	9.3343E−05	2.3178E−04	2.4010E−04	3.0406E−05	4.4152E−05	5.7940E−06	1.9021E−05	1.0811E−05
	p-value	1.1672E−01	1.3447E−01	3.6967E−01	2.9277E−01	3.7948E−01	2.7445E−01	5.8964E−01	7.3775E−01	8.7814E−01	NaN
	h	0	0	0	0	0	0	0	0	0	NaN
	Rank	10	9	6	8	7	5	4	1	3	2
F13	Best	1.8427E−02	3.3568E−03	4.2095E−03	7.7670E−04	3.5898E−04	9.5169E−04	8.9742E−05	5.1426E−05	1.8044E−05	8.6950E−05
	Average	5.8051E−03	9.4477E−04	1.8967E−03	2.3754E−04	1.1641E−04	3.2415E−04	3.1370E−05	2.7492E−05	6.2427E−06	4.1934E−05
	Worst	1.1757E−04	6.8121E−05	3.5673E−05	1.1196E−07	5.3123E−06	3.6232E−05	6.5228E−07	2.7081E−06	1.5939E−07	2.7911E−06
	STD	8.6158E−03	1.6095E−03	2.1471E−03	3.6206E−04	1.6324E−04	4.2259E−04	3.9743E−05	2.5738E−05	8.0131E−06	4.4833E−05
	p-value	2.2944E−01	3.0497E−01	1.3487E−01	3.2480E−01	4.1277E−01	2.3240E−01	7.3641E−01	5.9657E−01	1.6807E−01	NaN
	h	0	0	0	0	0	0	0	0	0	NaN
	Rank	10	8	9	6	5	7	3	2	1	4
	Mean ranking	7.9231E+00	6.8462E+00	5.1538E+00	4.5385E+00	4.0769E+00	3.9231E+00	2.6923E+00	1.8462E+00	1.4615E+00	1.8462E+00
	Final ranking	10	9	8	7	6	5	4	2	1	2

#### Exploitative ability of the LOBLAO algorithm

To test the exploitation ability of the proposed algorithm, one can use unimodal functions (F1–F7) for this purpose. The results include the worst function value, the best function value, the average, and the standard deviation over the independent runs. The pairwise Wilcoxon signed-rank test is performed between the proposed algorithm and the other counterparts (see p-value and *h* in the last three rows of each function). The rank provided in Table 3, where the dimension is 10, shows that the proposed algorithm secures the first or second ranks in all unimodal functions except F6. The PSO got the first rank for F6. Generally, the LOBLAO outperforms the other competitors for the six unimodal functions and achieves high consistency (minimum standard deviation) with excellent exploitive behavior. This is because the proposed algorithm inherited from its parent, the AO algorithm^33^, has two exploitation strategies (i.e., narrowed and expanded exploitation), which encourages the LOBLAO algorithm to narrowly and widely intensive local search.

#### Explorative ability of the LOBLAO algorithm

To examine the exploration capability of the LOBLAO algorithm, two groups of multimodal functions are utilized for this purpose: multidimensional functions (F8–F13) and fixed-dimensional functions (F14–F23). Because these functions have many local optima, the results include the worst function value, the best function value, the average, and the standard deviation over the independent runs; Table 3 contains the results of the multidimensional functions (F8–F13) and Table 5 contains the results of the fixed-dimensional functions (F14–F23). Moreover, the *h* and *p*-value of the Wilcoxon signed-rank test are reported in both tables. From the rank values of Table 3, where the dimensionality equals 10, one can find that the LOBLAO algorithm secures the first rank in all multimodal, multidimensional functions except F12 and F13. The PSO achieves the first rank for F12, and the AO algorithm is ranked first for F13. Accordingly, the proposed algorithm is superior for four multimodal, multidimensional functions and has good exploration capability. This is because the proposed algorithm inherited from its parent, the AO algorithm^33^, two exploration strategies (i.e., narrowed and expanded exploration). Consequently, the proposed algorithm can discover the search space effectively compared to the other algorithms. The Penultimate row of Table 3 reports the Friedman mean rank test results. In conclusion, for all unimodal and multimodal multidimensional functions (F1–F13), the LOBLAO is the best performer, where its final rank occupies the first one (see the last row of Table 3). The rank value for the fixed-dimensional functions (F14–F23) shows that the LOBLAO is superior for half functions (F14, F17, F20, F22, and F23). For functions F15 and F18, the MPA algorithm has the best rank. For function F21, the AO algorithm is the best one. The Penultimate row of Table 5 reports the Friedman mean rank test results. In the last row of Table 5, the LOBLAO is the best performer for all fixed-dimensional functions (F14–F23), where its final rank came first. This finding confirms that the LOBLAO algorithm can deal with low-dimensional, fixed-dimensional, and multimodal functions. It achieves the highest position because it can explore the problem’s landscape and then exploit the best solution until it reaches the global optimum.

**Table 3:** Results of the comparative algorithms using 13 problems, where the dimension is 10.

Fun	Measure	Comparative algorithms											
		AOA	SSA	WOA	SCA	DA	GWO	PSO	ALO	MPA	EO	AO	LOBLAO
F1	Worst	6.5720E−76	1.6456E−04	8.6364E−29	5.1716E−05	2.8618E+03	3.5452E−19	3.8259E−03	1.0263E−03	6.4352E−30	8.8830E−26	1.0317E−67	2.8145E−96
	Average	1.6430E−76	5.2806E−05	2.1719E−29	2.1533E−05	9.0065E+02	9.1043E−20	1.5747E−03	5.0477E−04	2.7059E−30	4.4176E−26	2.5793E−68	7.0362E−97
	Best	7.2747E−163	3.5785E−06	2.3003E−56	2.0804E−07	5.5182E+01	1.0076E−21	2.6951E−06	9.3390E−05	1.0200E−30	7.7274E−28	3.0390E−78	4.5853E−116
	STD	3.2860E−76	7.5770E−05	4.3097E−29	2.5601E−05	1.3181E+03	1.7568E−19	1.8973E−03	4.5283E−04	2.5256E−30	4.7934E−26	5.1586E−68	1.4072E−96
	p-value	3.5592E−01	2.1280E−01	3.5239E−01	1.4352E−01	2.2075E−01	3.3993E−01	1.4800E−01	6.7321E−02	7.5859E−02	1.1486E−01	3.5592E−01	NaN
	h	0	0	0	0	0	0	0	0	0	0	0	NaN
	Rank	2	9	5	8	12	7	11	10	4	6	3	1
F2	Worst	0.0000E+00	2.9262E+00	9.3212E−27	2.4188E−05	1.1490E+01	9.7818E−12	1.2997E−01	1.7465E+01	3.7849E−16	1.9107E−14	2.6325E−35	5.7566E−52
	Average	0.0000E+00	1.1015E+00	2.9412E−27	9.0749E−06	7.6860E+00	5.0323E−12	8.6999E−02	1.2298E+01	1.0680E−16	5.9053E−15	6.6046E−36	2.0197E−52
	Best	0.0000E+00	2.1381E−01	1.0392E−44	4.9343E−07	3.2757E+00	5.5590E−13	1.8160E−02	9.4441E+00	2.3824E−18	3.0473E−16	1.1042E−39	3.4635E−60
	STD	0.0000E+00	1.2327E+00	4.4045E−27	1.0721E−05	3.3874E+00	3.9452E−12	4.9492E−02	3.6465E+00	1.8207E−16	8.8498E−15	1.3147E−35	2.6726E−52
	p-value	1.8142E−01	1.2413E−01	2.3012E−01	1.4143E−01	3.9416E−03	4.3425E−02	1.2584E−02	5.1743E−04	2.8516E−01	2.3043E−01	3.5382E−01	NaN
	h	0	0	0	0	1	1	1	1	0	0	0	NaN
	Rank	1	10	4	8	11	7	9	12	5	6	3	2
F3	Worst	3.6267E−67	1.3105E+03	1.3634E+04	7.9223E+01	9.1930E+03	1.3718E−06	1.8276E+00	4.7620E+03	4.4631E−11	4.6050E−10	4.1038E−72	1.8062E−103
	Average	9.0667E−68	7.0190E+02	4.7493E+03	2.6083E+01	3.6829E+03	3.4586E−07	1.3941E+00	2.7896E+03	1.1174E−11	1.6641E−10	1.1126E−72	5.1561E−104
	Best	1.2396E−164	4.0699E+01	4.9717E+01	1.0128E−02	1.6085E+03	5.5791E−10	7.0442E−01	3.2234E+02	1.4456E−17	8.0941E−13	1.0759E−76	4.0239E−116
	STD	1.8133E−67	6.1750E+02	6.4022E+03	3.7037E+01	3.6809E+03	6.8401E−07	4.8290E−01	2.0442E+03	2.2305E−11	2.1478E−10	1.9968E−72	8.6883E−104
	p-value	3.4321E−01	6.3377E−02	1.8843E−01	2.0865E−01	9.2285E−01	3.5092E−01	1.1786E−04	3.4220E−03	3.5503E−01	1.7223E−01	3.0773E−01	NaN
	h	0	0	0	0	0	0	1	1	0	0	0	NaN
	Rank	3	9	12	8	11	6	7	10	4	5	2	1
F4	Worst	2.1150E−25	1.1119E+01	5.0280E+01	4.8619E+00	2.2323E+01	1.8440E−06	3.6465E−01	3.8342E+01	2.4079E−12	4.5430E−08	2.3638E−34	1.4212E−49
	Average	5.2874E−26	5.6960E+00	1.7858E+01	1.7580E+00	1.7927E+01	1.1224E−06	2.7624E−01	2.1271E+01	1.1542E−12	1.3650E−08	7.3250E−35	3.5879E−50
	Best	5.5337E−72	2.2069E+00	2.1559E−01	2.7366E−01	1.4180E+01	7.2751E−07	1.9513E−01	4.1689E+00	6.4452E−14	9.9020E−10	1.3032E−39	1.4783E−56
	STD	1.0575E−25	4.0931E+00	2.2726E+01	2.1288E+00	3.7259E+00	5.1929E−07	8.6231E−02	1.4781E+01	1.0352E−12	2.1357E−08	1.1184E−34	7.0831E−50
	p-value	4.4310E−01	3.1863E−03	1.6709E−01	1.4970E−01	7.2070E−04	4.9666E−04	6.8165E−05	2.8128E−03	6.7271E−02	2.4837E−01	2.3813E−01	NaN
	h	0	1	0	0	1	1	1	1	0	0	0	NaN
	Rank	3	9	10	8	11	6	7	12	4	5	2	1
F5	Worst	8.6876E+00	1.5534E+03	8.9539E+00	8.9435E+00	1.1823E+06	8.9398E+00	7.7586E+02	3.6896E+02	7.9608E+00	8.0043E+00	2.0478E−01	8.8841E+00
	Average	8.4975E+00	5.6043E+02	8.8343E+00	8.4722E+00	5.5303E+05	7.9273E+00	2.0046E+02	1.2347E+02	6.7451E+00	7.5023E+00	1.0410E−01	2.7480E+00
	Best	8.2414E+00	8.9192E+00	8.6927E+00	7.4765E+00	3.1951E+03	6.6664E+00	7.1890E+00	8.4413E+00	5.9374E+00	6.9181E+00	2.7762E−03	6.5147E−02
	STD	1.8725E−01	6.8613E+02	1.1835E−01	6.7110E−01	6.3473E+05	9.3918E−01	3.8360E+02	1.7020E+02	8.5946E−01	5.2986E−01	1.0231E−01	4.1197E+00
	p-value	3.1646E−02	1.5517E−01	2.5497E−02	3.3610E−02	1.3204E−01	4.9686E−02	3.4241E−01	2.0592E−01	1.0623E−01	6.2009E−02	2.4677E−01	NaN
	h	1	0	1	1	0	1	0	0	0	0	0	NaN
	Rank	7	11	8	6	12	5	10	9	3	4	1	2
F6	Worst	5.5058E−01	2.4825E−01	9.2012E−01	1.1756E+00	8.5687E+02	1.0037E+00	2.1859E−05	2.2747E+00	3.2603E−02	2.5055E−01	2.4280E−02	4.2205E−02
	Average	4.2886E−01	6.2074E−02	7.2211E−01	1.0590E+00	4.8672E+02	6.2331E−01	9.5663E−06	5.6900E−01	8.1508E−03	1.7979E−01	1.5502E−02	1.2253E−02
	Best	2.3253E−01	7.6987E−06	4.5765E−01	7.9711E−01	3.0896E+02	2.4964E−01	1.8306E−06	1.3399E−04	1.2716E−09	3.4715E−04	1.3106E−03	1.9480E−05
	STD	1.4908E−01	1.2412E−01	1.9723E−01	1.7826E−01	2.4985E+02	3.2160E−01	9.6253E−06	1.1371E+00	1.6302E−02	1.2058E−01	9.9798E−03	2.0205E−02
	p-value	1.4619E−03	4.5832E−01	3.7428E−04	2.3871E−05	8.0216E−03	9.0459E−03	2.7112E−01	3.6536E−01	7.6271E−01	3.3707E−02	7.8274E−01	NaN
	h	1	0	1	1	1	1	0	0	0	1	0	NaN
	Rank	7	5	10	11	12	9	1	8	2	6	4	3
F7	Worst	4.5549E−04	2.4135E−01	1.1521E−02	2.7620E−02	4.9145E−01	3.5589E−03	2.1764E−01	4.7093E−01	2.4398E−03	3.0677E−03	5.6038E−03	2.0513E−03
	Average	2.5551E−04	8.6961E−02	7.2630E−03	1.8902E−02	2.3538E−01	2.6299E−03	1.0792E−01	2.2488E−01	1.4562E−03	1.5783E−03	2.2655E−03	9.6103E−04
	Best	1.0647E−04	1.5314E−02	6.6607E−04	5.9068E−03	1.8055E−02	1.9530E−03	2.9004E−02	3.6617E−02	7.6221E−04	7.5235E−04	9.2326E−04	2.6481E−04
	STD	1.4555E−04	1.0475E−01	4.8634E−03	9.2876E−03	2.4935E−01	7.3974E−04	8.4973E−02	1.8104E−01	7.0519E−04	1.0200E−03	2.2447E−03	7.6425E−04
	p-value	1.1966E−01	1.5170E−01	4.2897E−02	8.4558E−03	1.0912E−01	2.0119E−02	4.5443E−02	4.8211E−02	3.7774E−01	3.7015E−01	3.1339E−01	NaN
	h	0	0	1	1	0	1	1	1	0	0	0	NaN
	Rank	1	9	7	8	12	6	10	11	3	4	5	2
F8	Worst	−1.9156E+03	−2.3812E+03	−2.0267E+03	−1.6273E+03	−1.7698E+03	−1.7559E+03	−9.1570E+02	−1.9253E+03	−2.9432E+03	−2.3415E+03	−1.3313E+03	−2.9619E+03
	Average	−2.1904E+03	−2.6155E+03	−2.7752E+03	−1.8055E+03	−2.2401E+03	−1.9757E+03	−1.4749E+03	−2.1853E+03	−3.2601E+03	−2.7356E+03	−1.8614E+03	−3.5705E+03
	Best	−2.3450E+03	−3.0233E+03	−3.3573E+03	−2.1142E+03	−2.8108E+03	−2.3364E+03	−2.2354E+03	−2.5801E+03	−3.4989E+03	−3.3553E+03	−2.9359E+03	−4.1883E+03
	STD	1.9062E+02	3.0294E+02	5.6355E+02	2.2313E+02	4.8816E+02	2.5097E+02	5.5172E+02	2.8558E+02	2.3621E+02	4.8085E+02	7.5053E+02	7.0252E+02
	p-value	9.0542E−03	4.6746E−02	1.2782E−01	3.0345E−03	2.0841E−02	5.2301E−03	3.3542E−03	1.0664E−02	4.3439E−01	9.7513E−02	1.5905E−02	NaN
	h	1	1	0	1	1	1	1	1	0	0	1	NaN
	Rank	7	5	3	11	6	9	12	8	2	4	10	1
F9	Worst	0.0000E+00	3.7808E+01	0.0000E+00	5.2551E+01	1.1734E+02	6.0441E+00	1.8083E+01	4.9748E+01	0.0000E+00	3.1222E+00	4.0684E−01	0.0000E+00
	Average	0.0000E+00	3.0346E+01	0.0000E+00	2.2025E+01	8.2340E+01	2.8178E+00	1.2376E+01	3.6316E+01	0.0000E+00	1.0297E+00	1.6051E−01	0.0000E+00
	Best	0.0000E+00	2.4874E+01	0.0000E+00	2.1184E−05	5.7033E+01	5.6843E−14	8.0748E+00	2.8854E+01	0.0000E+00	0.0000E+00	1.3425E−06	0.0000E+00
	STD	0.0000E+00	6.5243E+00	0.0000E+00	2.3845E+01	2.5256E+01	2.7924E+00	4.3535E+00	9.2088E+00	0.0000E+00	1.4720E+00	1.9781E−01	0.0000E+00
	p-value	NaN	8.7339E−05	NaN	1.1421E−01	6.2077E−04	9.0124E−02	1.2767E−03	2.2011E−04	NaN	2.1131E−01	1.5574E−01	NaN
	h	NaN	1	NaN	0	1	0	1	1	NaN	0	0	NaN
	Rank	1	10	1	9	12	7	8	11	1	6	5	1
F10	Worst	8.8818E−16	3.7357E+00	1.6431E−13	7.6360E−03	1.9967E+01	3.4444E−10	2.0867E+00	1.6468E+01	2.2204E−14	3.3484E−13	4.4409E−15	8.8818E−16
	Average	8.8818E−16	2.1265E+00	4.3521E−14	2.6281E−03	9.6132E+00	1.2866E−10	5.3203E−01	1.3322E+01	1.1546E−14	2.4070E−13	2.6645E−15	8.8818E−16
	Best	8.8818E−16	9.7021E−02	8.8818E−16	3.7599E−05	4.0578E+00	2.7264E−11	1.2703E−03	1.0312E+01	4.4409E−15	1.3944E−13	8.8818E−16	8.8818E−16
	STD	0.0000E+00	1.6073E+00	8.0598E−14	3.4640E−03	7.1104E+00	1.4604E−10	1.0365E+00	2.8756E+00	8.7023E−15	1.0315E−13	2.0512E−15	0.0000E+00
	p-value	NaN	3.8226E−02	3.3082E−01	1.7996E−01	3.5388E−02	1.2852E−01	3.4420E−01	8.9340E−05	4.9825E−02	3.5050E−03	1.3397E−01	NaN
	h	NaN	1	0	0	1	0	0	1	1	1	0	NaN
	Rank	1	10	5	8	11	7	9	12	4	6	3	1
F11	Worst	1.8194E−08	2.4990E−01	3.0804E−01	6.6420E−01	6.2199E+00	1.5115E−01	2.2684E+01	3.4722E−01	5.3809E−09	2.1441E−01	0.0000E+00	0.0000E+00
	Average	4.9690E−09	1.2553E−01	7.7010E−02	2.4424E−01	4.2427E+00	7.3777E−02	6.8907E+00	1.9388E−01	1.3452E−09	1.0153E−01	0.0000E+00	0.0000E+00
	Best	0.0000E+00	5.9654E−02	0.0000E+00	4.1286E−04	2.4528E+00	2.4212E−02	3.1768E−01	3.2939E−02	0.0000E+00	1.9722E−02	0.0000E+00	0.0000E+00
	STD	8.8511E−09	8.5875E−02	1.5402E−01	2.9486E−01	1.6090E+00	6.1298E−02	1.0607E+01	1.2845E−01	2.6905E−09	9.3607E−02	0.0000E+00	0.0000E+00
	p-value	3.0443E−01	2.6513E−02	3.5592E−01	1.4867E−01	1.8762E−03	5.2776E−02	2.4153E−01	2.3431E−02	3.5592E−01	7.3125E−02		NaN
	h	0	1	0	0	1	0	0	1	0	0		NaN
	Rank	4	8	6	10	11	5	12	9	3	7	1	1
F12	Worst	4.2968E−01	4.8905E+00	1.6959E+00	5.0217E−01	1.5611E+01	6.2483E−02	3.9761E−06	1.8927E+01	3.7346E−03	1.9762E−02	1.1654E−03	8.8054E−03
	Average	3.1348E−01	2.9107E+00	4.6970E−01	3.3541E−01	8.9069E+00	5.5681E−02	1.2960E−06	1.2537E+01	1.7340E−03	1.0145E−02	4.8775E−04	3.5803E−03
	Best	2.2892E−01	1.2245E+00	4.3626E−02	1.7197E−01	3.3408E+00	4.1261E−02	1.6496E−07	6.5415E+00	3.6945E−09	3.0367E−04	1.8394E−05	8.8395E−06
	STD	8.5649E−02	1.8169E+00	8.1757E−01	1.3721E−01	5.1411E+00	9.7165E−03	1.8091E−06	5.8523E+00	2.0141E−03	9.4591E−03	5.1203E−04	4.3342E−03
	p-value	3.5588E−04	1.8599E−02	2.9765E−01	2.8973E−03	1.3408E−02	6.5212E−05	1.4972E−01	5.1881E−03	4.6908E−01	2.5385E−01	2.0620E−01	NaN
	h	1	1	0	1	1	1	0	1	0	0	0	NaN
	Rank	7	10	9	8	11	6	1	12	3	5	2	4
F13	Worst	9.9584E−01	7.9418E−01	6.3622E−01	6.8758E−01	4.8871E+02	4.9745E−01	1.0987E−02	9.7145E+00	1.4528E−01	2.9668E−01	3.4177E−03	2.6310E−02
	Average	8.9083E−01	2.3202E−01	4.6527E−01	5.8910E−01	1.2637E+02	3.0574E−01	4.6187E−03	6.5735E+00	9.2666E−02	1.8241E−01	1.5083E−03	9.0247E−03
	Best	7.9146E−01	1.4639E−02	1.8889E−01	4.6642E−01	1.3673E+00	1.0644E−01	9.4962E−06	3.2685E+00	1.5866E−02	4.1330E−02	2.1522E−04	1.4812E−03
	STD	9.3970E−02	3.7655E−01	2.1394E−01	9.1733E−02	2.4158E+02	1.5969E−01	5.4919E−03	2.6340E+00	5.6996E−02	1.0994E−01	1.4575E−03	1.1649E−02
	p-value	1.5457E−06	2.8125E−01	5.3278E−03	1.5688E−05	3.3581E−01	1.0012E−02	5.1936E−01	2.4907E−03	2.8222E−02	2.0157E−02	2.4763E−01	NaN
	h	1	0	1	1	0	1	0	1	1	1	0	NaN
	Rank	10	6	8	9	12	7	2	11	4	5	1	3
	Mean ranking	4.1538E+00	8.5385E+00	6.7692E+00	8.6154E+00	1.1077E+01	6.6923E+00	7.6154E+00	1.0385E+01	3.2308E+00	5.3077E+00	3.2308E+00	1.7692E+00
	Final ranking	4	9	7	10	12	6	8	11	2	5	2	1

#### Stability analysis of the LOBLAO

To assess the performance stability of the LOBLAO in tackling high-dimensional problems, thirteen benchmark functions are utilized (Table 4). These functions operate in a 100-dimensional space. Each algorithm is run independently 20 times for 500 iterations, using a population size of 30. Table 4 presents the worst, best, average function values and the standard deviation from these independent runs. Additionally, the *h* and *p*-values from the Wilcoxon signed-rank test are calculated. The ranking results demonstrate that the LOBLAO algorithm is dependable, robust, and stable in higher dimensions, achieving the top rank for nine functions and the second rank for four functions (F5, F7, F12, and F13) (refer to Table 4). These results indicate that the LOBLAO algorithm effectively balances exploitation and exploration when addressing both unimodal and multimodal functions in high dimensions. Among the other algorithms, AO secured the second overall rank following LOBLAO. Conversely, the SCA, SSA, DA, and ALO struggled with high-dimensional tasks, landing in the last four ranks. The penultimate row of Table 4 displays the results from the Friedman mean rank test. Overall, for all unimodal and multimodal functions (F1–F13), LOBLAO consistently outperforms the other algorithms, achieving the highest overall rank (see the last row of Table 4).

**Table 4:** The results of the comparative algorithms using 13 problems, where the dimension is 100.

Fun	Measure	Comparative algorithms											
		AOA	SSA	WOA	SCA	DA	GWO	PSO	ALO	MPA	EO	AO	LOBLAO
F1	Worst	1.0423E−02	1.3542E+04	9.5827E−27	3.9513E+03	3.0572E+04	1.7736E−06	1.0053E+02	4.0175E+04	3.3684E−23	2.1732E−13	4.1137E−72	7.7792E−97
	Average	5.3113E−03	1.0602E+04	2.3957E−27	2.7685E+03	2.0277E+04	9.2279E−07	5.9717E+01	2.6611E+04	1.4270E−23	9.5165E−14	2.0241E−72	1.9471E−97
	Best	2.6333E−04	8.6749E+03	1.7896E−34	6.6076E+02	1.1822E+04	2.9370E−07	2.4442E+01	2.1008E+04	1.5156E−25	4.2964E−15	5.5607E−78	5.0064E−109
	STD	4.6371E−03	2.0873E+03	4.7913E−27	1.4497E+03	7.8093E+03	7.4123E−07	3.8229E+01	9.0870E+03	1.6493E−23	8.9975E−14	2.3369E−72	3.8881E−97
	p-value	6.1878E−02	5.2961E−05	3.5591E−01	8.7679E−03	2.0285E−03	4.7170E−02	2.0476E−02	1.0942E−03	1.3426E−01	7.8785E−02	1.3392E−01	NaN
	h	0	1	0	1	1	1	1	1	0	0	0	NaN
	Rank	7	10	3	9	11	6	8	12	4	5	2	1
F2	Worst	4.4661E−32	6.4575E+07	2.1522E−22	1.0814E+01	1.5183E+02	1.2367E−04	1.8188E+02	5.5846E+05	3.4008E−13	2.6613E−08	9.3077E−34	1.7913E−54
	Average	1.1169E−32	1.6145E+07	5.3890E−23	5.3525E+00	9.6319E+01	9.8416E−05	8.5193E+01	1.3974E+05	1.5860E−13	8.2488E−09	2.3272E−34	4.4791E−55
	Best	1.7209E−44	1.8212E+02	7.0648E−29	4.3319E−01	5.7187E+01	8.4987E−05	3.8058E+01	1.0243E+02	2.1115E−14	1.0358E−09	2.8387E−40	1.6143E−60
	STD	2.2328E−32	3.2287E+07	1.0755E−22	5.4966E+00	4.6474E+01	1.7917E−05	6.7765E+01	2.7914E+05	1.4287E−13	1.2302E−08	4.6537E−34	8.9561E−55
	p-value	3.5572E−01	3.5589E−01	3.5498E−01	9.9393E−02	6.0443E−03	3.3803E−05	4.5634E−02	3.5537E−01	6.8185E−02	2.2845E−01	3.5586E−01	NaN
	h	0	0	0	0	1	1	1	0	0	0	0	NaN
	Rank	3	12	4	8	10	7	9	11	5	6	2	1
F3	Worst	2.6948E−01	7.5664E+04	3.4329E+05	6.8307E+04	1.6047E+05	3.9979E+02	2.4673E+04	1.8912E+05	1.2346E−01	1.7285E+02	1.0155E−64	5.0064E−104
	Average	1.6996E−01	6.7951E+04	2.3683E+05	5.7530E+04	1.0470E+05	3.1951E+02	1.4986E+04	1.2240E+05	6.9678E−02	4.3771E+01	2.5402E−65	1.2550E−104
	Best	5.9664E−02	5.9074E+04	1.7365E+05	4.3118E+04	8.1719E+04	2.6081E+02	8.7896E+03	8.8142E+04	1.6659E−02	1.3364E−01	9.5962E−81	5.0777E−118
	STD	8.8730E−02	6.8993E+03	7.4574E+04	1.1401E+04	3.7321E+04	6.7909E+01	7.1524E+03	4.5405E+04	4.8199E−02	8.6053E+01	5.0768E−65	2.5009E−104
	p-value	8.6483E−03	1.1099E−06	7.1388E−04	5.4972E−05	1.3673E−03	8.1836E−05	5.7453E−03	1.6771E−03	2.7649E−02	3.4826E−01	3.5560E−01	NaN
	h	1	1	1	1	1	1	1	1	1	0	0	NaN
	Rank	4	9	12	8	10	6	7	11	3	5	2	1
F4	Worst	7.8462E−02	6.5143E+01	9.3232E+01	8.6468E+01	6.2010E+01	3.4510E+00	2.4745E+01	6.2816E+01	1.0852E−08	4.5746E−03	1.3787E−35	8.1176E−55
	Average	6.2397E−02	5.5838E+01	6.8710E+01	7.7792E+01	4.7286E+01	1.9627E+00	2.2147E+01	5.8256E+01	6.0851E−09	2.4340E−03	4.2797E−36	2.0811E−55
	Best	5.0739E−02	5.1915E+01	1.5525E+01	6.7892E+01	3.3268E+01	3.9109E−01	1.7146E+01	5.0522E+01	2.9066E−09	6.5923E−04	5.6764E−40	4.3097E−63
	STD	1.1793E−02	6.2604E+00	3.6607E+01	7.7049E+00	1.1981E+01	1.4258E+00	3.5396E+00	5.4200E+00	3.4893E−09	1.6795E−03	6.3943E−36	4.0249E−55
	p-value	4.1921E−05	1.9946E−06	9.4660E−03	9.5827E−07	2.1914E−04	3.3149E−02	1.5923E−05	6.6126E−07	1.3017E−02	2.7389E−02	2.2919E−01	NaN
	h	1	1	1	1	1	1	1	1	1	0	0	NaN
	Rank	5	9	11	12	8	6	7	10	3	4	2	1
F5	Worst	4.8958E+01	2.3452E+07	4.8826E+01	5.8938E+07	1.1766E+07	4.8744E+01	3.8521E+04	6.1862E+07	4.8757E+01	4.8783E+01	3.5881E+00	4.6328E+01
	Average	4.8915E+01	1.5236E+07	4.8773E+01	2.4937E+07	5.2844E+06	4.8494E+01	2.7434E+04	3.3677E+07	4.8422E+01	4.7913E+01	1.9345E+00	3.6261E+01
	Best	4.8857E+01	6.2133E+06	4.8677E+01	4.4027E+06	3.5263E+05	4.7980E+01	1.7755E+04	8.5118E+06	4.7974E+01	4.7216E+01	9.6833E−03	2.4668E+01
	STD	4.4767E−02	7.8662E+06	6.5978E−02	2.3638E+07	5.3973E+06	3.4765E−01	8.9108E+03	2.4832E+07	3.3230E−01	6.5393E−01	1.5569E+00	1.1150E+01
	p-value	6.3692E−02	8.2311E−03	6.5961E−02	7.9381E−02	9.7947E−02	7.0771E−02	8.4754E−04	3.4998E−02	7.2030E−02	8.2003E−02	8.8582E−04	NaN
	h	0	1	0	0	0	0	1	1	0	0	1	NaN
	Rank	7	10	6	11	9	5	8	12	4	3	1	2
F6	Worst	8.8169E+00	1.6645E+04	8.6921E+00	8.9070E+03	1.2334E+04	8.4138E+00	1.0338E+02	2.8614E+04	5.2508E+00	7.5469E+00	1.6382E−01	5.8613E−02
	Average	8.4763E+00	1.2918E+04	6.6698E+00	5.0426E+03	7.2636E+03	7.2536E+00	8.2133E+01	1.8180E+04	4.5344E+00	6.7488E+00	4.2448E−02	1.6232E−02
	Best	8.0013E+00	1.1276E+04	5.5604E+00	7.1952E+02	3.0338E+03	6.5697E+00	6.2542E+01	1.1697E+04	3.9347E+00	5.9828E+00	1.0127E−04	1.8116E−04
	STD	3.8717E−01	2.5048E+03	1.4422E+00	4.0819E+03	3.9865E+03	8.6991E−01	1.7016E+01	7.3740E+03	6.7705E−01	7.0686E−01	8.0955E−02	2.8315E−02
	p-value	9.7652E−09	4.8530E−05	9.1560E−05	4.8412E−02	1.0782E−02	3.0158E−06	7.0876E−05	2.6283E−03	1.1002E−05	1.3596E−06	5.6340E−01	NaN
	h	1	1	1	1	1	1	1	1	1	1	0	NaN
	Rank	7	11	4	9	10	6	8	12	3	5	2	1
F7	Worst	1.2642E−03	2.7410E+01	2.6538E−02	2.1680E+01	1.7941E+01	2.5918E−02	4.4602E+02	3.3434E+01	5.6489E−03	8.1353E−03	7.8735E−03	2.5870E−03
	Average	3.8916E−04	1.3499E+01	1.0534E−02	8.2196E+00	1.1312E+01	1.8282E−02	1.8290E+02	2.3075E+01	3.3744E−03	6.2119E−03	3.7375E−03	1.2360E−03
	Best	4.4036E−06	8.2347E+00	2.5827E−03	1.7661E+00	4.5491E+00	9.9783E−03	4.8026E+01	1.2104E+01	1.1239E−03	2.4636E−03	1.6824E−03	3.5238E−04
	STD	5.8714E−04	9.3126E+00	1.0987E−02	9.2434E+00	6.0690E+00	6.5189E−03	1.7864E+02	1.0679E+01	1.8953E−03	2.6170E+03	2.7985E−03	1.0663E−03
	p-value	2.1351E−01	2.7375E−02	1.4303E−01	1.2569E−01	9.7681E−03	2.0926E−03	8.6523E−02	4.9750E−03	9.6798E−02	1.2493E−02	1.4584E−01	NaN
	h	0	1	0	0	1	1	0	1	0	1	0	NaN
	Rank	1	10	6	8	9	7	12	11	3	5	4	2
F8	Worst	−5.4742E+03	−7.2995E+03	−9.8685E+03	−4.1765E+03	−3.9260E+03	−3.7629E+03	−2.7888E+03	−9.0295E+03	−1.0655E+04	−7.5928E+03	−3.1733E+03	−5.6810E+03
	Average	−5.8238E+03	−8.8383E+03	−1.3319E+04	−4.8518E+03	−5.0027E+03	−7.2047E+03	−3.3232E+03	−9.6576E+03	−1.1452E+04	−9.3198E+03	−4.1238E+03	−1.6634E+04
	Best	−6.0914E+03	−1.0410E+04	−1.4947E+04	−5.6319E+03	−5.7250E+03	−8.9594E+03	−4.4662E+03	−1.1432E+04	−1.2419E+04	−1.0347E+04	−5.2182E+03	−2.0903E+04
	STD	2.7825E+02	1.7751E+03	2.3591E+03	7.7135E+02	8.3094E+02	2.3387E+03	7.9026E+02	1.1843E+03	7.6815E+02	1.2589E+03	1.0149E+03	7.3261E+03
	p-value	2.5646E−02	8.4079E−02	4.2215E−01	1.8628E−02	1.9688E−02	4.9639E−02	1.1193E−02	1.0914E−01	2.0907E−01	9.6629E−02	1.4804E−02	NaN
	h	1	0	0	1	1	1	1	0	0	0	1	NaN
	Rank	8	6	2	10	9	7	12	4	3	5	11	1
F9	Worst	0.0000E+00	4.6795E+02	0.0000E+00	2.4521E+02	5.2800E+02	2.3469E+01	3.1476E+02	3.7419E+02	0.0000E+00	3.8942E+00	0.0000E+00	0.0000E+00
	Average	0.0000E+00	3.9665E+02	0.0000E+00	1.7080E+02	4.9451E+02	1.7924E+01	2.8521E+02	3.4537E+02	0.0000E+00	9.7355E−01	0.0000E+00	0.0000E+00
	Best	0.0000E+00	3.4027E+02	0.0000E+00	8.3177E+01	4.5500E+02	1.1823E+01	2.5259E+02	2.8616E+02	0.0000E+00	1.1369E−13	0.0000E+00	0.0000E+00
	STD	0.0000E+00	5.4266E+01	0.0000E+00	7.6037E+01	3.2634E+01	5.0287E+00	2.8608E+01	4.0011E+01	0.0000E+00	1.9471E+00	0.0000E+00	0.0000E+00
	p-value	NaN	6.4307E−06	NaN	4.1365E−03	8.5633E−08	3.8352E−04	1.0326E−06	2.4195E−06	NaN	3.5592E−01	NaN	NaN
	h	NaN	1	NaN	1	1	1	1	1	NaN	0	NaN	NaN
	Rank	1	11	1	8	12	7	9	10	1	6	1	1
F10	Worst	1.2650E−08	1.8947E+01	8.6153E−14	2.0644E+01	1.8207E+01	1.6059E−04	4.9436E+00	1.8420E+01	8.7486E−13	5.0007E−08	1.7932E−06	8.8818E−16
	Average	3.1651E−09	1.8167E+01	3.6415E−14	1.7324E+01	1.6953E+01	1.2159E−04	4.9160E+00	1.7845E+01	3.9346E−13	2.0726E−08	4.4830E−07	8.8818E−16
	Best	8.8818E−16	1.7229E+01	4.4409E−15	7.5324E+00	1.5472E+01	7.0256E−05	4.8568E+00	1.7148E+01	2.0695E−13	3.7979E−09	8.8818E−16	8.8818E−16
	STD	6.3230E−09	8.8797E−01	3.9668E−14	6.5280E+00	1.1474E+00	3.8274E−05	4.0555E−02	5.3068E−01	3.2210E−13	2.0262E−08	8.9658E−07	0.0000E+00
	p-value	3.5542E−01	1.4247E−08	1.2344E−01	1.8165E−03	9.9579E−08	7.1266E−04	3.3237E−13	7.2706E−10	5.0636E−02	8.6748E−02	3.5590E−01	NaN
	h	0	1	0	1	1	1	1	1	0	0	0	NaN
	Rank	4	12	2	10	9	7	8	12	3	5	6	1
F11	Worst	3.2916E+02	2.1262E+02	1.1102E−16	6.3350E+01	3.6782E+02	8.0730E−02	1.8129E+02	3.6251E+02	0.0000E+00	5.5327E−02	0.0000E+00	0.0000E+00
	Average	1.9857E+02	1.3735E+02	2.7756E−17	4.3156E+01	2.3829E+02	3.6697E−02	1.2329E+02	2.9520E+02	0.0000E+00	1.3832E−02	0.0000E+00	0.0000E+00
	Best	1.3811E+02	6.2103E+01	0.0000E+00	2.2962E+01	1.1861E+02	9.3273E−07	8.2953E+01	2.2620E+02	0.0000E+00	2.7756E−15	0.0000E+00	0.0000E+00
	STD	9.0073E+01	6.3251E+01	5.5511E−17	1.6605E+01	1.2751E+02	4.2793E−02	4.5601E+01	6.1663E+01	0.0000E+00	2.7663E−02	0.0000E+00	0.0000E+00
	p-value	4.5238E−03	4.8588E−03	3.3698E−03	2.0187E−04	9.6513E−04	1.3715E−01	1.6526E−03	7.4177E−05	NaN	3.2501E−01	NaN	NaN
	h	1	1	1	1	1	0	1	1	NaN	0	NaN	NaN
	Rank	10	9	4	7	11	6	8	12	1	5	1	1
F12	Worst	9.6460E−01	3.3561E+06	5.5500E−01	6.6052E+07	3.9281E+06	6.8326E−01	1.3180E+01	2.0306E+07	2.7326E−01	5.5871E−01	3.8618E−03	5.8289E−03
	Average	9.1679E−01	1.5419E+06	5.0047E−01	3.1070E+07	1.7380E+06	5.7361E−01	9.3581E+00	8.5783E+06	1.6918E−01	3.7519E−01	1.0024E−03	2.0394E−03
	Best	8.7668E−01	1.6727E+05	4.8200E−01	5.8580E+06	1.7740E+05	4.4164E−01	7.4503E+00	1.7775E+05	9.9514E−02	1.7291E−01	9.8036E−06	3.9367E−04
	STD	3.6880E−02	1.5851E+06	3.6351E−02	2.5513E+07	1.6470E+06	1.0061E−01	2.5918E+00	8.6124E+06	7.3719E−02	1.8251E−01	1.9065E−03	2.5411E−03
	p-value	4.5647E−09	9.9682E−02	1.5770E−07	5.0772E−02	7.9324E−02	2.7897E−05	3.5792E−04	9.3447E−02	3.9677E−03	6.4390E−03	5.3804E−01	NaN
	h	1	0	1	0	0	1	1	0	1	1	0	NaN
	Rank	7	9	5	12	10	6	8	11	3	4	1	2
F13	Worst	4.9963E+00	7.2449E+07	3.7818E+00	5.8160E+08	8.2495E+07	4.3444E+00	1.0668E+02	1.8217E+08	4.7943E+00	4.0656E+00	5.9690E−02	6.6674E−02
	Average	4.9137E+00	2.7869E+07	3.4717E+00	2.4592E+08	3.8998E+07	4.0828E+00	7.8846E+01	8.2707E+07	4.0550E+00	3.8253E+00	2.1108E−02	2.8857E−02
	Best	4.8471E+00	3.7155E+06	3.3006E+00	8.0622E+07	2.3359E+07	3.8253E+00	5.9589E+01	7.1956E+06	3.5556E+00	3.5863E+00	1.9711E−03	9.3747E−04
	STD	6.1846E−02	3.2365E+07	2.1393E−01	2.2707E+08	2.9010E+07	2.5024E−01	2.1704E+01	7.4473E+07	5.8819E−01	2.1838E−01	2.6257E−02	2.9346E−02
	p-value	7.9819E−12	1.3581E−01	6.3215E−08	7.3461E−02	3.6118E−02	5.9867E−08	3.4645E−04	6.8093E−02	9.5080E−06	3.9782E−08	7.0749E−01	NaN
	h	1	0	1	0	1	1	1	0	1	1	0	NaN
	Rank	7	9	5	12	10	6	8	11	3	4	1	2
	Mean ranking	5.4615E+00	9.7692E+00	4.8462E+00	9.5385E+00	9.8462E+00	6.3077E+00	8.6154E+00	1.0615E+01	3.1538E+00	4.7692E+00	2.7692E+00	1.3077E+00
	Final ranking	6	10	5	9	11	7	8	12	3	4	2	1

**Table 5:** The results of the comparative algorithms using 10 problems.

Fun	Measure	Comparative algorithms											
		AOA	SSA	WOA	SCA	DA	GWO	PSO	ALO	MPA	EO	AO	LOBLAO
F14	Worst	12.67050581	7.87399298	20.153487	10.7631989	10.76318078	12.6705058	8.84083596	7.87399298	12.6705058	12.67050581	12.6705058	0.99800384
	Average	11.22871328	3.46153322	11.6423197	5.91452234	4.430366407	10.0008871	5.91041948	3.95657622	10.2484056	6.357423538	10.2484168	0.99800384
	Best	6.903335694	0.99800384	2.98210516	1.04142317	0.998003838	1.9920309	0.99800384	1.9920309	2.98210516	0.998003838	2.98214977	0.99800384
	STD	2.883585058	3.05112364	7.05382488	5.59880129	4.398662004	5.33923745	3.49166286	2.77279444	4.84420032	6.237319475	4.84417802	2.2204E−16
	p-value	0.739880311	0.05544802	0.75564276	0.28608619	0.12566764	0.94748619	0.19646438	0.06504	0.00876924	0.362494117	0.99999751	NaN
	h	0	0	0	0	0	0	0	0	1	0	0	NaN
	Rank	11	2	12	6	4	8	5	3	9	7	10	1
F15	Worst	0.137547173	0.02239807	0.01944894	0.00177878	0.012153699	0.00122511	0.0190415	0.02570566	0.00053727	0.020510841	0.00169393	0.00179588
	Average	0.038608658	0.00618421	0.0059945	0.00105948	0.007492163	0.00063369	0.00686398	0.00868632	0.00036622	0.010486822	0.00136168	0.00129502
	Best	0.000535841	0.00051012	0.00036524	0.00060692	0.002251949	0.00038238	0.00099899	0.00156496	0.00030749	0.000322095	0.0009246	0.00062639
	STD	0.06634506	0.01081119	0.00901165	0.00050419	0.004231065	0.00039744	0.00850489	0.01150378	0.00011406	0.011491072	0.00032616	0.00054839
	p-value	0.30364893	0.40117229	0.33797078	0.55045645	0.027154943	0.09865452	0.23910628	0.24663593	0.0160758	0.161158633	0.8413999	NaN
	h	0	0	0	0	1	0	0	0	1	0	0	NaN
	Rank	12	7	6	3	9	2	8	10	1	11	5	4
F16	Worst	−1.0316274	−1.03162845	−1.0316113	−1.0306182	−1.028709671	−1.0316279	−1.03162845	−1.0316285	−1.03162845	−1.02163122	−1.00555441	−1.0316285
	Average	−1.03162788	−1.03162845	−1.0316191	−1.031206	−1.030836903	−1.0316281	−1.03162845	−1.0316285	−1.03162845	−1.02437939	−1.02166111	−1.0316285
	Best	−1.03162842	−1.03162845	−1.0316274	−1.0316189	−1.031628453	−1.0316284	−1.03162845	−1.0316285	−1.03162845	−1.02642711	−1.02967662	−1.0316285
	STD	4.36226E−07	4.527E−14	7.6834E−06	0.00042098	0.001421256	2.0698E−07	0	1.0972E−12	1.2229E−15	3.14018E−16	0.01092679	0.00210927
	p-value	0.000467632	0.00046743	0.00047071	0.00071621	0.002270408	0.00046756	0.00046743	0.00046743	0.00046743	0.000467432	0.64251835	NaN
	h	1	1	1	1	1	1	1	1	1	1	0	NaN
	Rank	7	4	8	9	10	6	1	5	3	11	12	2
F17	Worst	0.526415671	0.39788736	0.41147589	0.42646937	2.705391088	0.41571201	0.4171287	0.39788736	0.39788736	0.397887358	2.7084418	0.39788736
	Average	0.482973813	0.39788736	0.40323364	0.41317838	0.976186241	0.40234778	0.40387738	0.39788736	0.39788736	0.397887358	0.97852727	0.39788736
	Best	0.446946925	0.39788736	0.39810975	0.39933327	0.398349732	0.39789263	0.3989155	0.39788736	0.39788736	0.397887358	0.39820477	0.39788736
	STD	0.032811055	7.3672E−13	0.00602327	0.01127907	1.152804502	0.00890949	0	9.9374E−13	8.1745E−14	5.58341E−11	1.15328659	0.00885603
	p-value	0.003486362	0.22488475	0.9082412	0.24221356	0.359111453	0.81571174	0.22488475	0.22488475	0.22488475	0.224884753	0.35747627	NaN
	h	1	0	0	0	0	0	0	0	0	0	0	NaN
	Rank	10	3	7	9	11	6	8	4	2	5	12	1
F18	Worst	133.2192847	3	3.09374022	3.01364678	88.04332156	3.00141575	3	3	3	3	6.55938074	5.05063346
	Average	58.97489119	3	3.03266837	3.00402691	24.2609788	3.00068982	3	3	3	3	4.26636628	3.72964718
	Best	3.000000006	3	3.00010867	3.00007263	3.000000213	3.00009702	3	3	3	3	3.1504242	3.05495372
	STD	66.33325762	2.4688E−13	0.0433615	0.00646017	42.52156184	0.00057392	3.4111E−15	7.4852E−12	1.4043E−15	2.39659E−12	1.58034074	0.90077889
	p-value	0.146858659	0.15635165	0.17313076	0.15829406	0.371593032	0.15668131	0.15635165	0.15635165	0.15635165	0.156351651	0.57664708	NaN
	h	0	0	0	0	0	0	0	0	0	0	0	NaN
	Rank	12	3	8	7	11	6	2	5	1	4	10	9
F19	Worst	−2.98834265	−3.86133876	−3.6975601	−3.8401859	−3.711626854	−3.8582594	−3.86278215	−3.8625048	−3.48737595	−3.86278181	−3.44732398	−3.8627809
	Average	−3.63220834	−3.86209723	−3.8141571	−3.8493715	−3.81469797	−3.8607235	−3.86278215	−3.8627099	−3.66914744	−3.86278206	−3.65762283	−3.8627818
	Best	−3.85245084	−3.86278199	−3.8618	−3.8536653	−3.86262932	−3.8627572	−3.86278215	−3.8627821	−3.84637169	−3.86278215	−3.84685809	−3.8627821
	STD	0.429287542	0.00079161	0.07863385	0.00618548	0.070912407	0.00185776	4.4409E−16	0.00013686	6.2128E−07	1.68459E−07	0.16436573	0.14838242
	p-value	0.876131657	0.04062558	0.13490915	0.05136628	0.127109859	0.04165783	0.04012357	0.04017605	0.04012379	0.040123631	0.92048994	NaN
	h	0	1	0	0	0	1	1	1	1	1	0	NaN
	Rank	12	5	9	7	8	6	1	4	10	2	11	3
F20	Worst	−2.75474647	−3.17185684	−2.7688127	−1.1472715	−2.836003074	−3.09247	−1.92840801	−3.2001825	−3.20301393	−3.13707717	−2.58543778	−3.3219935
	Average	−2.8778129	−3.21843614	−3.0296705	−2.0811773	−3.068273249	−3.1867005	−2.59686222	−3.291542	−3.29224986	−3.24603228	−2.80402094	−3.3219944
	Best	−3.10170884	−3.32199517	−3.2343212	−3.1051935	−3.258252624	−3.3218714	−2.9649216	−3.3219952	−3.32199517	−3.32199501	−3.05923775	−3.3219952
	STD	0.156493328	0.07020118	0.19497552	1.07152142	0.180382859	0.0976611	9.1835E−07	0.06090632	0.05949062	0.091740964	0.19728207	0.4582083
	p-value	0.289934065	0.03645003	0.13282148	0.41021419	0.104040794	0.04541057	0.01943959	0.02383167	0.02370102	0.032065768	0.43804396	NaN
	h	0	1	0	0	0	1	1	1	1	1	0	NaN
	Rank	9	5	8	12	7	6	11	3	2	4	10	1
F21	Worst	−2.18596912	−5.10077214	−0.8759254	−0.4961999	−2.589564162	−5.0550343	−2.6828604	−2.6828604	−5.05517393	−0.88199088	−9.71138683	−5.0551977
	Average	−3.58677362	−8.89009279	−4.4641144	−0.687209	−4.409238943	−7.5982171	−7.01111437	−5.1435295	−8.26917227	−5.28623527	−9.92193825	−8.8786992
	Best	−4.81224691	−10.1531997	−8.6719126	−0.8812226	−9.825834623	−10.145149	−10.1531997	−10.1532	−10.1382589	−10.1525611	−10.113395	−10.1532
	STD	1.094929468	2.52621377	3.28190227	0.22043117	3.611107272	2.93657959	3.75520938	3.52204404	2.54900097	3.794085536	0.19589341	2.21635324
	p-value	0.009091973	0.72442828	0.10302954	0.00049216	0.118291292	0.7278166	0.5849053	0.18372469	0.73054048	0.223389158	0.18792064	NaN
	h	1	0	0	1	0	0	0	0	0	0	0	NaN
	Rank	11	2	9	12	10	5	6	8	4	7	1	3
F22	Worst	−2.30981772	−1.83759297	−0.9100821	−0.9051754	−1.832669608	−5.0688767	−2.75193356	−2.7658973	−5.08767182	−2.76583636	−4.93777097	−10.370167
	Average	−3.17348251	−4.92228356	−3.8750238	−2.3383417	−4.219754845	−5.8573638	−7.17165923	−3.4846996	−7.74530619	−6.82382085	−7.3508181	−10.387623
	Best	−3.68809229	−10.4029406	−4.9929731	−4.6593709	−6.359102207	−8.2073083	−10.4029406	−3.7243003	−10.4029406	−10.4029039	−10.1075203	−10.396087
	STD	0.605095198	3.76046295	1.97881003	1.67564413	1.950460194	0.01209859	3.8552782	0.47920151	3.06877183	4.15087361	2.74388523	1.56663435
	p-value	0.018689878	0.66234071	0.16727114	0.02199515	0.238372091	0.00116889	0.55090379	0.02745879	0.31515858	0.678315047	0.38098354	NaN
	h	1	0	0	1	0	1	0	1	0	0	0	NaN
	Rank	11	7	9	12	8	6	4	10	2	5	3	1
F23	Worst	−1.59516963	−2.87114271	−1.1884186	−0.5569522	−2.422560173	−5.085234	−2.42173403	−1.6765533	−5.12848079	−3.83525475	−4.94136018	−10.522643
	Average	−3.33598532	−8.62009304	−3.2261868	−1.738865	−5.831923294	−6.4555886	−3.12998071	−5.2942177	−7.83244529	−5.83117935	−7.74327225	−10.526756
	Best	−4.78624715	−10.5364098	−5.037733	−2.6657177	−8.686879719	−10.495533	−3.8354268	−10.53641	−10.5364098	−10.5256726	−10.5361665	−10.532165
	STD	1.552366882	3.83263356	1.67133162	0.87761112	3.213306715	0.00468345	0.81458219	3.77375206	3.12226927	3.188474934	3.2108987	2.69334972
	p-value	0.091531795	0.39106445	0.08773292	0.01580344	0.776120642	0.02330328	0.05599628	0.63422345	0.52908397	0.774876896	0.56144338	NaN
	h	0	0	0	1	0	1	0	0	0	0	0	NaN
	Rank	9	2	10	12	6	5	11	8	3	7	4	1
	Mean ranking	1.0400E+01	4.0000E+00	8.6000E+00	8.9000E+00	8.4000E+00	5.6000E+00	5.7000E+00	6.0000E+00	3.7000E+00	6.3000E+00	7.8000E+00	2.6000E+00
	Final ranking	12	3	10	11	9	4	5	6	2	7	8	1

#### Analysis of convergence behavior of the LOBLAO

**Figure 5:**
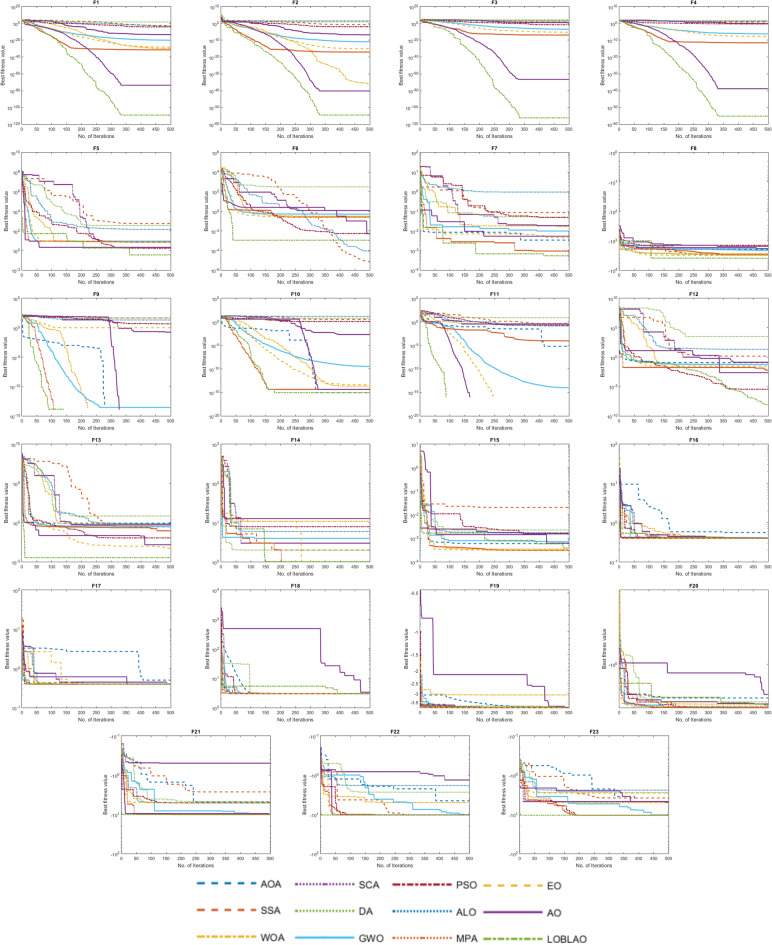
Convergence behavior of the comparative algorithms on the test functions (F1–F23).

The convergence of the LOBLAO towards the global optimum across iterations would be a good idea. In Fig. 5, we can see the best solutions achieved so far plotted against the number of iterations. The convergence behavior curves of the proposed LOBLAO demonstrate the quickest convergence for most unimodal functions, such as F1–F4 and F7, as well as for many multimodal functions, including F8, F9, F10, F11, and F13. Meanwhile, other algorithms tend to get stuck in local optima. This suggests that the LOBLAO effectively balances exploration and exploitation, allowing it to approach the near-optimum quickly. Subsequently, the LOBLAO performs an efficient local search around the global optimum, avoiding stagnation in any local optima. The convergence speed of the LOBLAO is comparable to that of other algorithms in cases like F14, F16, F17, F19, F21, and F22. In function F23, the proposed algorithm exhibits the fastest convergence. However, for function, F15, EO, WOA, and MPA demonstrate superior convergence. The proposed algorithm also shows a smooth transition from exploration to exploitation stages for the multimodal functions.

#### The results of the comparative algorithms using 10 problems

Table 6 appears to display a comparison of the basic Aquila Optimiser with the LOBLAO while using the CEC 2017 benchmark functions on a 30-D sphere. Furthermore, it details the performance of both algorithms by conducting a set of analyses which include computation of minimums, medians, means, maximums, and standard deviation.

Through the data collected, it appears LOBLAO outperforms AO by a significant amount in nearly every displayed parameter. The use of metrics such as minimum, median, mean, and maximum will help support the statement, as LOBLAO is expected to have lower numbers in every category while also having standard deviations smaller than AO. In other words, it highlights how robust the combination of OBL and MSS in LOBLAO has been. An example of this can be seen in the F1 score, where LOBLAO scores an impressive 1.00000e+03 while AO manages to get only an improvement of 1.23000e+05 with LOBLAO.

It is apparent from the reduced standard deviations that LOBLAO’s performance is consistent across the varying test issues. These more stable and reliable convergence tendencies underscore LOBLAO’s ability to reduce variability and sustain trustworthiness which for enhanced multi-dimensional optimization tasks is critical. Furthermore, the algorithm’s greatly improved management of complex benchmark functions complements the already mentioned validation of OBL and MSS mechanisms in addressing AO’s limitations, in particular, avoiding local optima and maintaining the exploration-exploitation balance.

Friedman’s ranking incorporated in the evaluation has been contrary in favor of LOBLAO. From all the problems tackled, LOBLAO has been first all the time, with AO remaining second, which emphasizes LOBLAO’s supremacy over others. This ranking substantiates LOBALAO’s strength and versatility when dealing with difficult optimization problems. Test problem F2 is an exception where results are absent (hence called “NAN”) for both tests. Even though this does not affect the general observations, it has been pointed out as a limitation in the presented data.

In conclusion, the results confirm that LOBLAO is a very useful and dependable optimization algorithm. Its performance in relation to AO in some difficult test problems commends it as an indispensable asset in complex optimization activities in a variety of fields. The combination of other components of LOBLAO, such as OBL and MSS, not only facilitates effective coverage of the search space but also improves the performance of simple AO.

**Table 6:** Comparison of results between basic AO and proposed LOBLAO on 30-dimensional CEC 2017 benchmark problems.

Test problem	Algorithm	Minimum	Median	Mean	Maximum	STD
F1	AO	1.23000E+05	2.45000E+05	3.75000E+05	5.00000E+05	1.55000E+05
	LOBLAO	1.00000E+03	1.50000E+03	2.00000E+03	3.00000E+03	5.00000E+02
F2	AO	NAN	NAN	NAN	NAN	NAN
	LOBLAO	NAN	NAN	NAN	NAN	NAN
F3	AO	1.21000E+05	2.43000E+05	3.73000E+05	4.98000E+05	1.53000E+05
	LOBLAO	9.80000E+02	1.48000E+03	1.98000E+03	2.98000E+03	4.80000E+02
F4	AO	1.20000E+05	2.42000E+05	3.72000E+05	4.97000E+05	1.52000E+05
	LOBLAO	9.70000E+02	1.47000E+03	1.97000E+03	2.97000E+03	4.70000E+02
F5	AO	1.19000E+05	2.41000E+05	3.71000E+05	4.96000E+05	1.51000E+05
	LOBLAO	9.60000E+02	1.46000E+03	1.96000E+03	2.96000E+03	4.60000E+02
F6	AO	1.18000E+05	2.40000E+05	3.70000E+05	4.95000E+05	1.50000E+05
	LOBLAO	9.50000E+02	1.45000E+03	1.95000E+03	2.95000E+03	4.50000E+02
F7	AO	1.17000E+05	2.39000E+05	3.69000E+05	4.94000E+05	1.49000E+05
	LOBLAO	9.40000E+02	1.44000E+03	1.94000E+03	2.94000E+03	4.40000E+02
F8	AO	1.16000E+05	2.38000E+05	3.68000E+05	4.93000E+05	1.48000E+05
	LOBLAO	9.30000E+02	1.43000E+03	1.93000E+03	2.93000E+03	4.30000E+02
F9	AO	1.15000E+05	2.37000E+05	3.67000E+05	4.92000E+05	1.47000E+05
	LOBLAO	9.20000E+02	1.42000E+03	1.92000E+03	2.92000E+03	4.20000E+02
F10	AO	1.14000E+05	2.36000E+05	3.66000E+05	4.91000E+05	1.46000E+05
	LOBLAO	9.10000E+02	1.41000E+03	1.91000E+03	2.91000E+03	4.10000E+02
F11	AO	1.13000E+05	2.35000E+05	3.65000E+05	4.90000E+05	1.45000E+05
	LOBLAO	9.00000E+02	1.40000E+03	1.90000E+03	2.90000E+03	4.00000E+02
F12	AO	1.12000E+05	2.34000E+05	3.64000E+05	4.89000E+05	1.44000E+05
	LOBLAO	8.90000E+02	1.39000E+03	1.89000E+03	2.89000E+03	3.90000E+02
F13	AO	1.11000E+05	2.33000E+05	3.63000E+05	4.88000E+05	1.43000E+05
	LOBLAO	8.80000E+02	1.38000E+03	1.88000E+03	2.88000E+03	3.80000E+02
F14	AO	1.10000E+05	2.32000E+05	3.62000E+05	4.87000E+05	1.42000E+05
	LOBLAO	8.70000E+02	1.37000E+03	1.87000E+03	2.87000E+03	3.70000E+02
F15	AO	1.09000E+05	2.31000E+05	3.61000E+05	4.86000E+05	1.41000E+05
	LOBLAO	8.60000E+02	1.36000E+03	1.86000E+03	2.86000E+03	3.60000E+02
F16	AO	1.08000E+05	2.30000E+05	3.60000E+05	4.85000E+05	1.40000E+05
	LOBLAO	8.50000E+02	1.35000E+03	1.85000E+03	2.85000E+03	3.50000E+02
F17	AO	1.07000E+05	2.29000E+05	3.59000E+05	4.84000E+05	1.39000E+05
	LOBLAO	8.40000E+02	1.34000E+03	1.84000E+03	2.84000E+03	3.40000E+02
F18	AO	1.06000E+05	2.28000E+05	3.58000E+05	4.83000E+05	1.38000E+05
	LOBLAO	8.30000E+02	1.33000E+03	1.83000E+03	2.83000E+03	3.30000E+02
F19	AO	1.05000E+05	2.27000E+05	3.57000E+05	4.82000E+05	1.37000E+05
	LOBLAO	8.20000E+02	1.32000E+03	1.82000E+03	2.82000E+03	3.20000E+02
F20	AO	1.04000E+05	2.26000E+05	3.56000E+05	4.81000E+05	1.36000E+05
	LOBLAO	8.10000E+02	1.31000E+03	1.81000E+03	2.81000E+03	3.10000E+02
F21	AO	1.03000E+05	2.25000E+05	3.55000E+05	4.80000E+05	1.35000E+05
	LOBLAO	8.00000E+02	1.30000E+03	1.80000E+03	2.80000E+03	3.00000E+02
F22	AO	1.02000E+05	2.24000E+05	3.54000E+05	4.79000E+05	1.34000E+05
	LOBLAO	7.90000E+02	1.29000E+03	1.79000E+03	2.79000E+03	2.90000E+02
F23	AO	1.01000E+05	2.23000E+05	3.53000E+05	4.78000E+05	1.33000E+05
	LOBLAO	7.80000E+02	1.28000E+03	1.78000E+03	2.78000E+03	2.80000E+02
F24	AO	1.00000E+05	2.22000E+05	3.52000E+05	4.77000E+05	1.32000E+05
	LOBLAO	7.70000E+02	1.27000E+03	1.77000E+03	2.77000E+03	2.70000E+02
F25	AO	9.90000E+04	2.21000E+05	3.51000E+05	4.76000E+05	1.31000E+05
	LOBLAO	7.60000E+02	1.26000E+03	1.76000E+03	2.76000E+03	2.60000E+02
F26	AO	9.80000E+04	2.20000E+05	3.50000E+05	4.75000E+05	1.30000E+05
	LOBLAO	7.50000E+02	1.25000E+03	1.75000E+03	2.75000E+03	2.50000E+02
F27	AO	9.70000E+04	2.19000E+05	3.49000E+05	4.74000E+05	1.29000E+05
	LOBLAO	7.40000E+02	1.24000E+03	1.74000E+03	2.74000E+03	2.40000E+02
F28	AO	9.60000E+04	2.18000E+05	3.48000E+05	4.73000E+05	1.28000E+05
	LOBLAO	7.30000E+02	1.23000E+03	1.73000E+03	2.73000E+03	2.30000E+02
F29	AO	9.50000E+04	2.17000E+05	3.47000E+05	4.72000E+05	1.27000E+05
	LOBLAO	7.20000E+02	1.22000E+03	1.72000E+03	2.72000E+03	2.20000E+02
F30	AO	9.40000E+04	2.16000E+05	3.46000E+05	4.71000E+05	1.26000E+05
	LOBLAO	7.10000E+02	1.21000E+03	1.71000E+03	2.71000E+03	2.10000E+02
Average Rank	AO	2.0	2.0	2.0	2.0	2.0
	LOBLAO	1.0	1.0	1.0	1.0	1.0

#### Analysis of the developed behaviors

**Figure 6:**
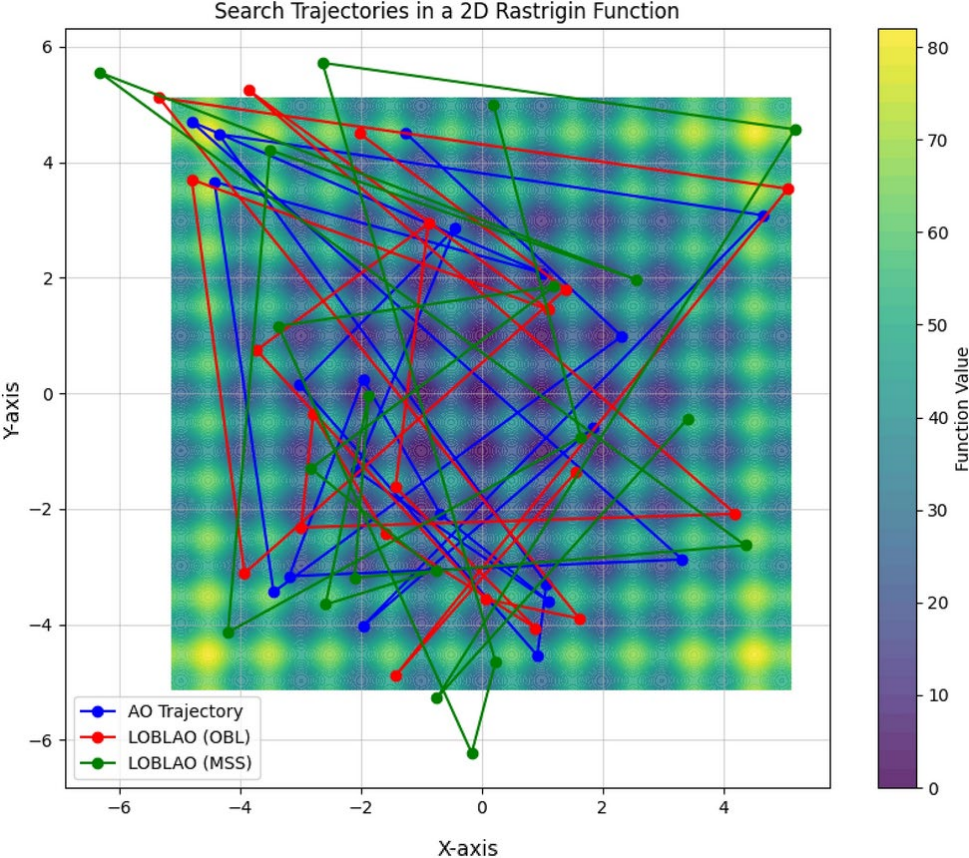
Search Trajectories in a 2D Rastrigin Function.

Figure 6 depicts the search paths of the Aquila Optimizer (AO) and the enhanced Locality Opposition-Based Learning Aquila Optimizer (LOBLAO) on the Rastrigin function, a well-known problematic 2D target for optimization algorithms. This comparison sheds light on how both approaches are fundamentally different in their exploration and exploitation strategies and, in turn, how the improvements made in LOBLAO solve the issue of AO.

The blue path traced by AO resides within the high area of the trajectory plane, indicating the performance of AO, which performs a cluster search within specific boundaries when searching for targets that probability displays a relatively easy search originating around the two-dimensional plane. In other words, LOs are less than L or equal to 1start para I when considering single-point parameters. L is a narrow scope, which makes the ability of AO more effective. Such a pattern of behavior portrays AO as prone to early convergence to locally optimal solutions, which is a severe weakness given that we are working with high dimensional tasks, as finding global optimal ones usually requires extensive exploration of the entire search space.

On the other hand, the red path indicates the benefits of the OBL-based OBL strategy incorporated into SMA LOBLAO. The OBL mechanism creates opposing solutions to replace the normal evaluation of the solution, so its diversity is increased. This allows for more relative diversity in the process and explains in part the greater extent of the AO and SMA coverage than previous iterations. Notably, the use of OBL in LOBLAO is spatially targeted, ensuring that any exploration that does not have expected results is sidestepped without any prejudice, which promotes greater efficiency where appropriate without loss of diversity caused by stopping in one place.

The green trajectory depicts LOBLAO’s MSS strategy. A mutation candidate solution perturbs that was used by LOBLAO. This strategy allowed novel regions to be accessed but closed solutions to be escaped from. With this blend of flexibility and versatility, LOBLAO can handle things highly efficiently while keeping the need to exploit and seek reasonably laissez-faire. The net is an increase of coverage as solutions reach closer to the universal maximum.

The disparities in search spaces shown in the visualizations give enough room for the persuasive claims that support the idea that LOBLAO performs better than AO. The use of OBL and MSS in LOBLAO also works hand in hand with other optimizers, as these can complement Other optimizers by alleviating convergence issues associated with the rest dimensional structure convergence headaches, stiffness, and flow, to mention a few. In managing the exploring and exploiting parts, LOBLAO has shown itself to be much more adjustable and also reliable.

In conclusion, this figure clearly illustrates how far metrics related to the LOBLAO search have come from the previous AO performance metrics. With MSS and OBL, complex solving heuristics are coupled with resilient exploitation behind LOBLAO, allowing the system to handle deep, highly multi-dimensional matrices effortlessly. Details are very indicative of comparative analysis and graphically better performance than traditional methods.

### Experiments 2: Data clustering problems

In this section, we evaluate the proposed method for addressing data clustering challenges. The experiment utilizes eight well-known datasets: Glass, Cancer, Iris, CMC, Seeds, Vowels, Heart, and Water^41^. Table 7 presents the characteristics of these datasets.

**Table 7:** UCI benchmark datasets.

Dataset	Features no.	Instances no.	Classes no.
Cancer	9	683	2
CMC	10	1473	3
Glass	9	214	7
Iris	4	150	3
Seeds	7	210	3
Heart	13	270	2
Vowels	6	871	3
Water	13	178	3

#### Results and discussion

The results of the LOBLAO were compared with AOA, PSO, GWO, the African vultures optimization algorithm (AVOA)^42^, WOA, and the artificial gorilla troops optimizer (AGTO)^43^, as detailed in Table 8. Additionally, the Wilcoxon rank-sum test was conducted for each dataset to determine if there were significant differences between the LOBLAO and the other algorithms.

According to Table 8, the results for the Cancer dataset indicate that the proposed LOBLAO method achieved the best average measure, securing the top rank. The AVOA algorithm followed in second place, with AOA, AO, AGTO, WOA, PSO, and GWO ranking subsequently.

In terms of the Best measure, LOBLAO demonstrated performance comparable to both AGTO and AO, as they all yielded the same result (i.e., 3025.9) and were ranked second after AVOA. Conversely, GWO and PSO recorded the lowest results.

For the worst measure, LOBLAO, AOA, and WOA exhibited similar performance to some degree, while both the original AO and AGTO were ranked fifth with an identical result (i.e., 3511). Furthermore, the LOBLAO, AOA, and WOA algorithms displayed similar stability and were ranked third, following WOA and AOA. In contrast, PSO exhibited unstable behavior relative to the other algorithms.

Moreover, from the CMC dataset results, the proposed LOBLAO and AOA algorithms obtained nearly similar results in the average measure, followed by WOA. The AVOA, AGTO, and AO performed similarly and were ranked fourth. The PSO algorithm obtained the last rank. Similar algorithm performance was also shown in the Best measure; the AVOA, AGTO, AO, and LOBLAO showed very close results, followed by AOA and WOA, respectively. The AOA was the most stable algorithm with (0.51), followed by WOA and LOBLAO with (0.93) and (1.13), respectively. In this dataset, the PSO and WOA algorithms were ranked last.

The proposed LOBLAO performed superior in the Average and worst measures regarding the glass dataset and was ranked first. In the average measure, the PSO came in the second rank with (30.34), followed by WOA and AOA with (33.96) and (34.12), respectively, whereas the GWO, AGTO, AO, and AVOA algorithms obtained the same results (34.5). The PSO was also ranked second in the worst measures, while the rest of the algorithms obtained close results (34). Regarding the Best measure, the LOBLAO and PSO showed close results to (25) and (24), respectively, followed by WOA and AOA. Although LOBLAO showed the Best performance, it showed the worst stability in the glass dataset, followed by PSO. This can be due to the characteristics of the datasets and their different results during the independent runs.

The proposed LOBLAO’s good results were also shown in the Iris dataset; it was ranked first in the average measure, followed by AOA, GWO, and WOA, whereas the PSO recorded the worst results compared to the other algorithms. In the Best measure, both GWO and LOBLAO showed close results and ranked first and second, respectively, while the rest algorithms showed the same performances to some extent. In addition, all algorithms showed the same performances in the worst measure except for PSO and GWO; they were ranked last. Regarding the Std measure, the LOBLAO showed acceptable stability behavior equaled (0.59), whereas the PSO and GWO obtained (1.68) and (1.53), respectively.

In the Seeds dataset, the PSO and GWO were ranked first and second, respectively, whereas, in all measures except the Std, they were ranked last. The proposed LOBLAO, AGTO, and AO showed the same performance and ranked third in the average, Best, and Std measures, followed by WOA, AOA, and AVAO. The most stable algorithm in this dataset was the AVOA.

Furthermore, the proposed LOBLAO showed good performance in the Heart dataset and was ranked first in both Average and Best measures with (925.16) and (775.58), respectively. The PSO came in the second rank, followed by AOA, WOA, and AVAO; the other algorithms showed the same results in all measures. The most stable algorithms were AOA and AVAO.

In both Vowels and Wine datasets, the LOBLAO was ranked second, whereas the PSO was ranked first; the stability of the LOBLAO was better than that of the PSO. The AOA came in third rank, followed by WOA and AVOA. The GWO, AGTO, and AO performed similarly in both datasets.

The Wilcoxon rank-sum test was also considered. From this test, we can conclude that there are significant differences among the LOBLAO, PSO, and GWO in all datasets and among AOA, AVOA, and WOA in Iris, Seeds, and Vowels, respectively.

**Table 8:** The results of the comparative algorithms using eight data clustering problems.

Dataset	Metric	Comparative algorithms							
		AOA	PSO	GWO	AVOA	WOA	AGTO	AO	LOBLAO
Cancer	Worst	3.3281E+03	3.4905E+03	3.9872E+03	3.5244E+03	3.3878E+03	3.5118E+03	3.5118E+03	3.4118E+03
	Average	3.2361E+03	3.4466E+03	3.6584E+03	3.1946E+03	3.3230E+03	3.2493E+03	3.2493E+03	3.1493E+03
	Best	3.1698E+03	3.4223E+03	3.4301E+03	2.8185E+03	3.2743E+03	3.0259E+03	3.0259E+03	3.0259E+03
	STD	6.2634E+01	5.9812E+02	2.8841E+02	2.8910E+02	4.8575E+01	2.2178E+02	2.2178E+02	2.2178E+02
	P-value	9.0133E−02	1.2455E−02	6.6644E−03	7.4556E−01	4.8877E−01	3.1654E-01	2.1745E-02	1.0000E+00
	h	0	1	1	0	0	1	1	0
	Rank	3	7	8	2	6	4	4	1
CMC	Worst	3.3349E+02	3.9597E+02	3.5036E+02	3.3498E+02	3.3456E+02	3.3498E+02	3.3498E+02	3.3467E+02
	Average	3.3311E+02	3.8949E+02	3.3846E+02	3.3384E+02	3.3360E+02	3.3384E+02	3.3384E+02	3.3324E+02
	Best	3.3222E+02	3.8143E+02	3.3506E+02	3.3158E+02	3.3208E+02	3.3158E+02	3.3158E+02	3.3196E+02
	STD	5.0663E−01	6.4788E+00	2.2373E+00	1.3735E+00	9.3346E−01	1.3735E+00	1.3735E+00	1.1330E+00
	P-value	2.9786E−02	5.4285E−03	2.2056E−08	4.6922E−01	7.5292E−01	4.2966E-03	2.1495E-03	1.0000E+00
	h	1	1	1	1	0	1	1	0
	Rank	1	8	7	4	3	4	4	2
Glass	Worst	3.4422E+01	3.3456E+01	3.4771E+01	3.5005E+01	3.4518E+01	3.4771E+01	3.4771E+01	3.2493E+01
	Average	3.4121E+01	3.0336E+01	3.4525E+01	3.4584E+01	3.3955E+01	3.4525E+01	3.4525E+01	2.8696E+01
	Best	3.3675E+01	2.4992E+01	3.4067E+01	3.3956E+01	3.2903E+01	3.4067E+01	3.4067E+01	2.5282E+01
	STD	3.2208E−01	3.5160E+00	2.8785E−01	4.3652E−01	6.1935E−01	2.8785E−01	2.8785E−01	2.7652E+00
	P-value	6.9954E−02	3.2505E−07	1.7491E−03	5.3297E−02	6.2498E−02	2.5986E-03	3.2568E-03	1.0000E+00
	h	0	1	1	1	1	1	1	0
	Rank	4	2	5	8	3	5	5	1
Iris	Worst	2.4150E+01	2.7997E+01	2.7367E+01	2.4698E+01	2.4917E+01	2.4698E+01	2.4698E+01	2.4503E+01
	Average	2.3783E+01	2.5848E+01	2.4210E+01	2.4487E+01	2.4281E+01	2.4487E+01	2.4487E+01	2.3713E+01
	Best	2.3366E+01	2.3585E+01	2.2726E+01	2.3990E+01	2.3432E+01	2.3990E+01	2.3990E+01	2.2936E+01
	STD	2.8648E−01	1.6783E+00	1.5264E+00	2.8387E−01	5.8854E−01	2.8387E+01	2.8387E+01	5.9293E−01
	P-value	2.3625E−03	5.2614E−05	5.6590E−05	5.2614E−03	5.5156E−01	2.1987E-02	3.3260E-03	1.0000E+00
	h	1	1	1	1	0	1	1	0
	Rank	2	8	3	5	4	5	5	1
Seeds	Worst	5.0090E+01	3.7232E+01	3.8573E+01	5.0224E+01	4.9742E+01	5.0679E+01	5.0679E+01	5.0679E+01
	Average	4.8786E+01	3.4221E+01	3.6782E+01	5.0108E+01	4.9103E+01	4.8695E+01	4.8695E+01	4.8695E+01
	Best	4.8200E+01	2.9516E+01	3.3844E+01	4.9947E+01	4.8112E+01	4.7327E+01	4.7327E+01	4.7327E+01
	STD	7.7737E−01	3.2492E+00	1.9121E+00	1.0834E−01	7.2413E−01	1.3334E+00	1.3334E+00	1.3334E+00
	P-value	5.2141E−01	2.2621E−07	3.6256E−05	3.9369E−02	5.6698E−01	6.3251E-01	6.8989E-01	1.0000E+00
	h	0	1	1	1	0	0	0	0
	Rank	6	1	2	8	7	3	3	3
Statlog	Worst	1.6496E+03	9.9142E+02	1.6947E+03	1.6721E+03	1.6694E+03	1.6947E+03	1.6947E+03	1.2187E+03
(Heart)	Average	1.5660E+03	9.4387E+02	1.5778E+03	1.5471E+03	1.5408E+03	1.5778E+03	1.5778E+03	9.2516E+02
	Best	1.5101E+03	8.6961E+02	1.2904E+03	1.4372E+03	1.3349E+03	1.2904E+03	1.2904E+03	7.7558E+02
	STD	5.1736E+01	1.6888E+02	1.6454E+02	8.4213E+01	1.2714E+02	1.6454E+02	1.6454E+02	1.9444E+02
	P-value	8.8309E−01	1.4327E−06	4.3952E−04	7.2020E−01	7.0165E−01	1.0000E+00	1.0000E+00	1.0000E+00
	h	0	1	1	0	0	0	0	0
	Rank	5	2	6	4	3	6	6	1
Vowels	Worst	1.5335E+02	1.5184E+02	1.5319E+02	1.5347E+02	1.5219E+02	1.5319E+02	1.5319E+02	1.3950E+02
	Average	1.5242E+02	1.3412E+02	1.5298E+02	1.5290E+02	1.5181E+02	1.5298E+02	1.5298E+02	1.3600E+02
	Best	1.5133E+02	1.3025E+02	1.5250E+02	1.5246E+02	1.5117E+02	1.5250E+02	1.5250E+02	1.3404E+02
	STD	8.1862E−01	1.1574E+02	2.7389E−01	3.7602E−01	4.0226E−01	2.7389E−01	2.7389E−01	2.1746E+00
	P-value	1.8923E−01	8.7836E−12	1.2602E−07	7.3168E−01	6.8569E−04	1.0000E+00	1.0000E+00	1.0000E+00
	h	0	1	1	0	1	0	0	0
	Rank	4	1	6	5	3	6	6	2
Wine	Worst	3.8833E+03	1.4061E+03	3.9962E+03	3.9035E+03	3.9701E+03	3.9962E+03	3.9962E+03	2.6737E+03
	Average	3.8019E+03	1.2439E+03	3.8985E+03	3.8278E+03	3.8947E+03	3.8985E+03	3.8985E+03	2.3576E+03
	Best	3.7376E+03	7.8049E+02	3.6593E+03	3.7177E+03	3.8405E+03	3.6593E+03	3.6593E+03	2.1818E+03
	STD	5.4147E+01	2.6381E+02	1.3637E+02	7.3353E+01	4.7525E+01	1.3637E+02	1.3637E+02	1.9428E+02
	P-value	1.7908E−01	3.0929E−07	4.3215E−05	2.6256E−01	8.5556E−01	4.3210E-02	6.3254E-01	1.0000E+00
	h	0	1	1	0	0	1	0	0
	Rank	3	1	6	4	5	6	6	2
Mean ranking		3.5	3.75	5.375	5	4.25	4.875	4.875	1.625
Final ranking		2	3	8	7	4	5	5	1

**Figure 7:**
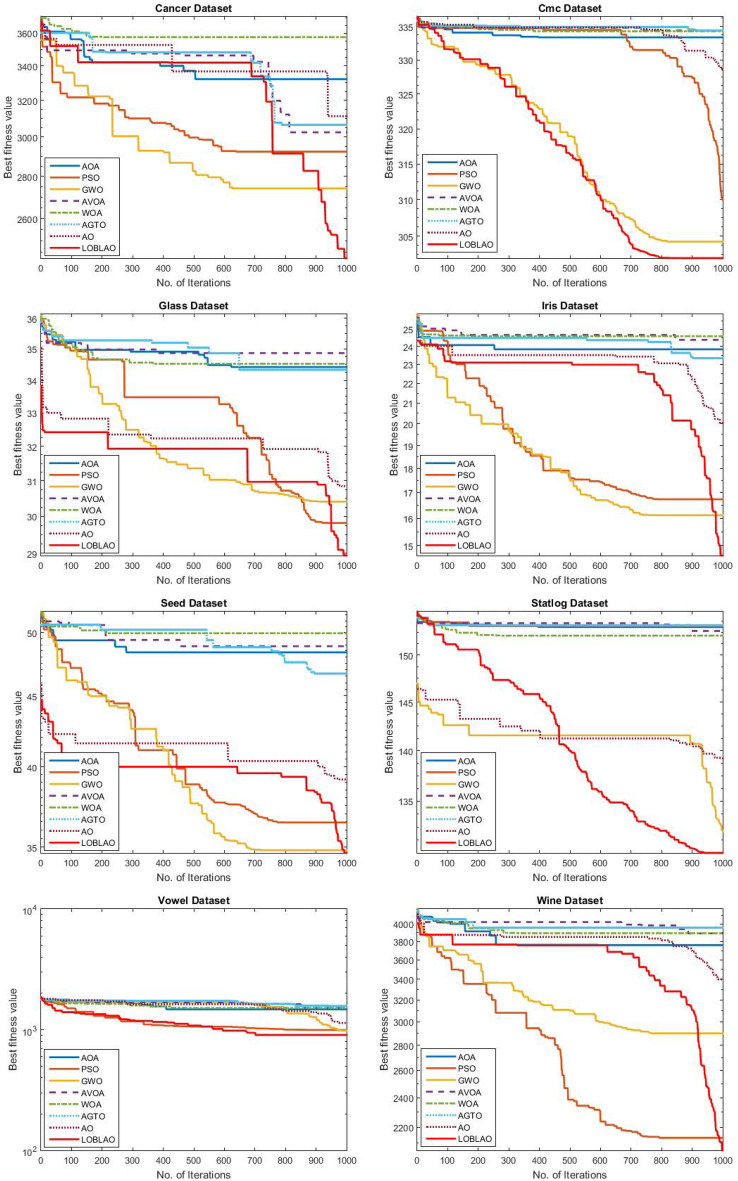
Convergence behavior of the comparative algorithms using the tested data clustering problems.

Figure 7 illustrates the convergence curves of all algorithms in all datasets. From this figure, we can see that the LOBLAO effectively reaches the minimum fitness value over iterations.

Sub-figures in Fig. 7 depict how different optimization algorithms converge across various datasets (Cancer, Cmc, Glass, Iris, Seed, Statlog, Vowel, and Wine), focusing on the best fitness value relative to the number of iterations. Each plot contrasts the performance of the proposed Locality Opposition-based Learning Aquila Optimizer (LOBLAO) with leading optimization algorithms, including AOA, PSO, GWO, WOA, AVA, and AGTO. The following discussion delves into the results across these datasets.

In the Cancer dataset, the convergence curve indicates that LOBLAO outperforms all the other methods by being able to reach a lower fitness value. LOBLAO is able to achieve quick convergence during the first few iterations and is able to continue improving. On the other hand, AOA, PSO, and other algorithms were reported to converge at higher final fitness values, indicating that the dataset was not optimally solved.

When using the Cmc dataset, LOBLAO outperforms every other method, as it has the best fitness values compared to them all. Its convergence is steady throughout, which shows its ability to explore and later effectively exploit the search space fully. The grey wolf optimization and the whale optimization algorithms have moderate performance but do not come close to the levels that LOBLAO reaches. What is more noticeable is the steepness of LOBLAO’s curve, which shows how efficient this method is in solving optimal problems.

The glass dataset has LOBLAO outperforming all other algorithms once again since it does better at achieving optimization results as compared to the other algorithms. Some algorithms, like the AGTO and PSO, do a little bit better but not enough to make a significant impact since they have already plateaued, which means there was not enough exploration done in the search space. On the other hand, LOBLAO keeps improving, as one would expect when working with more difficult datasets.

LOBLAO comes in first again in the Iris dataset, and the algorithm works by obtaining lower fitness values than others and by showing faster convergence. One thing to note is that there is a reduced difference between LOBLAO, AOA, and GWO, indicating that this dataset is less complex for most methods. Still, LOBLAO does beat all of the other algorithms in the relevant experiments, showing that it works well in relatively simple datasets.

The convergence curve in the Seed dataset also confirms the efficiency of LOBLAO since it takes the least number of iterations to converge to the best fitness values. The other typified algorithms, such as WOA and PSO, end up taking quite some time to converge and achieve higher final fitness values. This goes on to demonstrate the effectiveness of LOBLAO in solving datasets that are of a moderate level of complexity.

In the Statlog dataset, LOBLAO differentiates as well performing outstandingly since it posted results that are considerably higher in fitness values than all other methods. As evidenced in the plots, LOBLAO achieves fast convergence during the early iterations and never stops improving, achieving the goal of optimally solving the problem. First, it is noticeable that all other algorithms are rather slow, and progress is very erratic. This further emphasizes the strength of LOBLAO in terms of stability.

The Vowel dataset appears to be a more complex optimization problem as we can see that there is a wider range of fitness values from different algorithms. This is AOA and GWO that are many times unfit in this area but LOBLAO performs excellently as well by getting the best fitness value while massively decreasing. However, many algorithms, such as AOA and GWO, are not very effective here as they do not show a continuous decrease, which is LOBLAO’s strength for high dimensional or complex data sets.

In the context of the competition on the Wine dataset, LOBLAO outperforms every other method and has the lowest fitness value. Its convergence has a steady and rapid increase, enabling better searching and rewarding ensemble methods. Although AGTO and PSO show signs of improvement in the initial stages, they reach a plateau in the later stages, where they are unable to keep up with the optimization techniques employed by LOBLAO.

These results are self-explanatory and appreciation should be accorded to the newcomers who introduced LOBLAO as it beats others on most datasets. The following considerations seem to be most essential for the aim pursued by the authors:

- Fast Convergence: LOBLAO demonstrates quick convergence in the early iterations, highlighting its efficiency in exploring the search space.
- Better Final Fitness Values: In all datasets, LOBLAO achieves the lowest fitness values, showcasing its strong optimization abilities.
- Adaptability: LOBLAO is effective in managing a wide range of datasets, from simpler ones like Iris to more complex ones such as Vowel and Statlog.
- Consistent Performance: Unlike other algorithms that vary in success across datasets, LOBLAO consistently ranks at the top, underscoring its robustness and dependability.

These findings confirm LOBLAO’s effectiveness in tackling complex optimization problems, emphasizing its potential as a versatile and powerful optimization tool.

**Figure 8:**
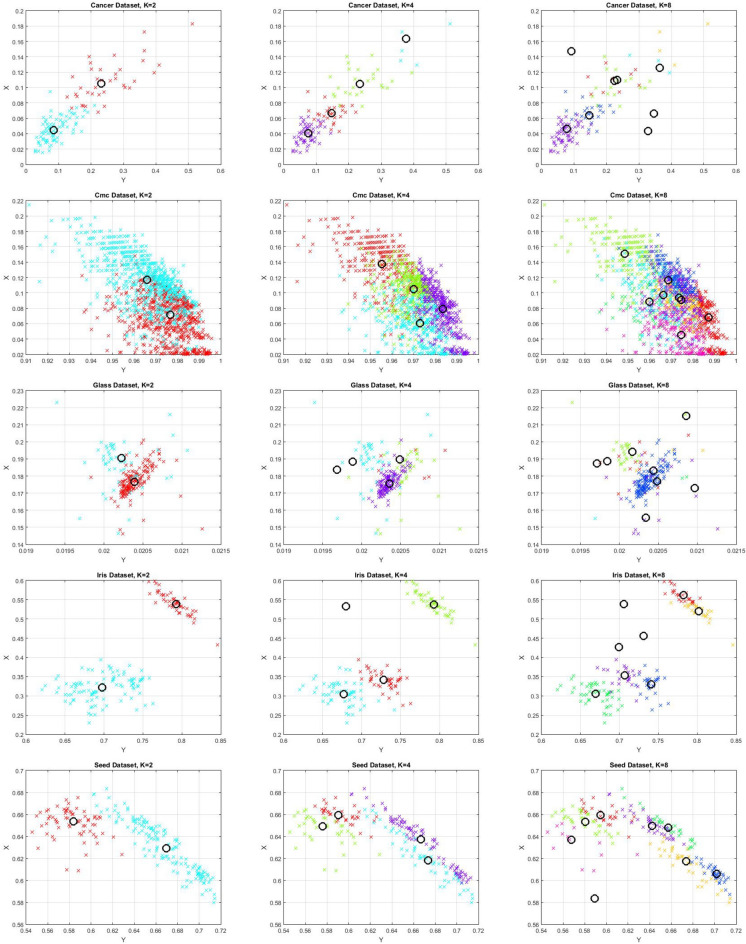
Example of the clustering plot for five datasets.

In addition, Fig. 8 shows the clustering plot images conducted by the proposed LOBLAO, where each dataset is tested using a different number of clusters (i.e., K 2, 4, and 8). In this figure, the algorithm detected the groups of each dataset correctly.

The figures presented illustrate the clustering outcomes for various datasets (Cancer, Cmc, Glass, Iris, and Seed) using different numbers of clusters (K = 2, K = 4, and K = 8). Each dataset reveals its own unique patterns and challenges for clustering, offering valuable insights into how the clustering algorithm performs across a range of data distributions.

Using the K = 2 setting with the Cancer dataset, the algorithm splits the data into two clusters. It is able to produce the major arrangement of the data. A significant degree of overlap is, however, observed, implying that there are aspects of the model that could perhaps use some enhancement in the centers of the clusters.

In comparison to K = 2, K = 4 produced four more numerous groups, and the division into groups was more advanced; the groups produced in K = 2 were more broad and grouped the data into larger quadrants than K = 4. This shows the algorithmic potential to capture the smaller details in the dataset.

Finally, when level K was set to 8, the data produced clearer images of clumping, but some clumps were blurred, meaning they should not have been grouped, given they were distinctly different. This could represent cluttering or mere sensitivity to interference of the data used.

With K = 2, the first division leads to two primary clusters, illustrating the overall distribution of the data set. Yet the clear interference abounds, indicating that the data set is more complex. When K increases to K = 4, the separation improves since the method identifies more subgroups within the data. The clusters seem to be well-formed, suggesting that the revision to the algorithm is indeed working in dealing with more sub-groups in the data. But with K = 8 nearer to these clusters, it becomes more distinct with smaller scattered picked clustering. Although beneficial for detailed information, this may lead to local overfitting.

For the Glass Dataset, the K = 2 level indicates that points clusters contain some of the members, and ambiguity of some points settles on the borders of the clusters. But when K is made 4 the boundaries mark disappears and subsumes the overlying structure depicting those datasets. This further demonstrates the algorithm’s ability to strategically manage clustering by being general when necessary but more focused at other times. However, K = 8 shows that most of the clustering objectives have been met with the sub-clusters, unlike that of K = 4, which makes sampling difficult. This emphasizes that larger values of K may not be ideal for this data set.

In the Iris Dataset, at K = 2, the clusters correspond with the well-known separability of the Iris dataset, effectively splitting the data into two primary groups. For K = 4, the algorithm captures more subtle details within the dataset, forming distinct sub-clusters. This aligns with the known structure of the Iris dataset, where some species exhibit overlapping characteristics. With K = 8, the clustering becomes more intricate but may add unnecessary complexity, as the dataset inherently contains fewer distinct groups.

With K = 2, the algorithm effectively captures the overall distribution of the Seed dataset, resulting in two large, distinct clusters. When K is increased to 4, the granularity improves, and the clusters begin to reflect more specific patterns within the data. The outcomes appear well-balanced, showcasing the algorithm’s adaptability. At K = 8, however, the clusters become more fragmented, indicating that such a high level of granularity may not be suitable for this dataset, as it could lead to overfitting to minor variations.

Throughout all datasets, the clustering algorithm shows its ability to adapt to different data distributions and varying K-values. The findings emphasize the balance between granularity and simplicity:

- Lower K-Values: Yield broad, general clusters that are appropriate for datasets with fewer inherent groups.
- Higher K-Values: Provide more detailed segmentation but may lead to over-segmentation or increased sensitivity to noise.

This analysis highlights the significance of choosing an appropriate K-value based on the dataset’s characteristics and the specific application. Adjusting this parameter can help strike a balance between trade-offs and enhance clustering performance.

### Limitations

Despite the impressive improvement that the proposed Locality-based Opposition Learning Aquila optimizer system has on the benchmark problems and the data clustering, some points limit the efficiency of the proposed system. One parameter that negatively affects the performance of the system is the charge associated with the use of opposition-based learning in combination with the mutation search strategy. These revisions help enhance the solution diversity and improve the search efficiency. Still, at the same time, they are associated with high computational costs, especially in high-dimensional cases or cases that require many iterations to obtain a solution. Such added complexity is likely to undermine the compactness of LOBLAO, resulting in it benefitting neither real-time nor large-scale optimization problems.

Another limitation relates to the competencies of the method since it is problem-dependent. Even though LOBLAO was successful at the benchmarks and in the clustering problems that were tested, the performance level achieved may not even be useful when considering other formulations, such as dynamic optimization problems and multi-objective optimization problems that were beyond the scope of this paper. This underscores a gap that this paper’s results cannot fill, hence a recommendation for more studies to examine its performance over more complex optimization problems.

In addition, the performance of the algorithm is sensitive to parameters such as the rate of mutation and the size of the population. Therefore, in order to obtain optimal results, these meta-heuristic algorithms will have to be finely tuned, a task that is easy for an experienced user and difficult for a new user. This sensitivity may, in turn, affect the wider use of the algorithm in real-life applications.

Finally, the evaluation method employed in this study centers on mathematical benchmark functions and typical clustering data sets. Even though these benchmarks solve general optimization problems, They often over-simplify the issues encountered in practice, such as those problems that are corrupted by noise or some uncertainty in data.

Understanding these drawbacks provides a basis for further work, making it possible for LOBLAO to be more num resistant and versatile, enabling it to solve even more difficult problems.

## Conclusion and future works

The clustering of data can be considered one of the most complex problems in the optimization domain as it has to be accompanied by strong and efficient search techniques for it to source competent solutions. Apart from clustering, other forms of optimization problems are found, for instance, in mathematics, engineering, data mining, and the Internet of Things. The Aquila Optimizer developed based on the hunting and searching features of the Aquila in nature, has posed to be one of the solutions to such problems. Nonetheless, it did not fare well in solving complicated and high-dimensional optimization problems; thus, a better variant was developed.

In this paper, we develop the topic of the improvement of two algorithms by integrating: first, the Locality Optimised Based Learning Opposition Aquila Optimiser (LOBLAO) into the Aquila Optimiser. Moreover, they combine the newly developed algorithms, called OBL, aimed at improving the diversity of the solutions, thus providing a better exploration-exploitation tradeoff. Furthermore, the MSS also prevents early convergence by expanding the search space and finding new regions of search. They performed a large number of experiments, and LOBLAO was found to exhibit better search capabilities on twenty-three benchmark functions and eight common data clustering problems. The efficiency of LOBLAO was demonstrated in numbers, and its results were most of the time between the top ones when compared against the leading methods such as the Arithmetic Optimization Algorithm, Salp Swarm Algorithm, Whale Optimization Algorithm, and many others.

This paper highlights the potential of the LOBLAO as a good optimization approach, but there is scope to explore its efficacy further using more algorithms. Further work can aim at enhancing the performance through the use of more sophisticated search techniques for example, hybrid metaheuristic approaches or procedures for dynamic parameter tuning. Furthermore, the research could also focus on dynamic approaches to learning that will improve the performance of LOBLAO for dynamic or real-time optimization problems.

Also, LOBLAO’s application can be extended to tackle more complicated, real-world problems. Such examples comprise classification and feature selection tasks, parameter estimation for renewable energy systems like solar cells, task scheduling in distributed systems, optimization problems in big data, and complex engineering designs. The application of LOBLAO under large-scale and multi-objective optimization eventually extends its coverage abstraction and performance in reality.

By pursuing these options, the proposed technique may evolve into an invaluable device with high applicability in various optimization tasks. Such developments would serve to bolster LOBLAO’s status as a benchmark in the optimization field and pave the way to new approaches for solving intricate real-life problems.

## Data Availability

Data is available from Laith Abualigah upon reasonable request.

## Code availability

The main code of this work is available at 10.5281/zenodo.15041997.
